# Synthesis, Characterization, and Biological Evaluation of Aliphatic‐Substituted Benzimidazole Derivatives: Induction of Apoptosis, Cell Cycle Arrest, and Molecular Docking in Breast Cancer Cells

**DOI:** 10.1002/ddr.70267

**Published:** 2026-03-29

**Authors:** Murat Keser, Çisil Çamlı Pulat, Harika Atmaca, Hakan Akgün, Canan Albay, Emre Menteşe, Hakan Bektaş, Suleyman Ilhan

**Affiliations:** ^1^ Department of Medical Oncology Medicana International İzmir Hospital İzmir Türkiye; ^2^ Applied Science Research Center Manisa Celal Bayar University Manisa Türkiye; ^3^ Department of Biology, Faculty of Engineering and Natural Sciences Manisa Celal Bayar University Manisa Türkiye; ^4^ Department of Chemistry, Faculty of Science and Art Giresun University Giresun Türkiye; ^5^ Department of Chemistry, Faculty of Science and Art Recep Tayyip Erdogan University Rize Türkiye

**Keywords:** aliphatic‐substituted benzimidazole, apoptosis, Bcl‐2, Bcl‐xL, breast cancer, CDK2, cell cycle arrest, cytotoxicity, molecular docking, multitarget anticancer agent

## Abstract

A new series of aliphatic‐substituted benzimidazole derivatives was synthesized and structurally characterized to evaluate their potential anticancer activity. Among the synthesized compounds, compound 4 exhibited the most potent cytotoxic effects against MCF‐7 and MDA‐MB‐231 breast cancer cell lines, with IC₅₀ values comparable to those of cisplatin, while displaying lower toxicity toward normal breast epithelial cells (MCF‐10A). Flow cytometric analysis revealed that treatment with compound 4 resulted in significant accumulation of cells in the S phase, indicating inhibition of DNA synthesis and replication. Furthermore, Annexin V/PI double‐staining analysis demonstrated a marked increase in both early and late apoptotic cell populations, confirming the activation of apoptotic pathways. Molecular docking studies supported these experimental findings by revealing strong interactions of compound 4 with key regulatory proteins involved in apoptosis and cell cycle progression, including Bcl‐2, Bcl‐xL, CDK2, and Cyclin E. The compound exhibited the highest binding affinity toward CDK2 (–164.055 kcal/mol), forming hydrogen bonds with critical residues (LEU134, ASP145, GLN131, and LYS33) within the ATP‐binding pocket, suggesting potential inhibition of kinase activity. Interactions with Bcl‐2 and Bcl‐xL occurred within the BH3‐binding grooves, which may impair their anti‐apoptotic functions and promote mitochondrial‐mediated apoptosis. Collectively, the in vitro and in silico results indicate that this newly synthesized benzimidazole derivative exerts its anticancer effects through a dual mechanism involving cell cycle arrest and apoptosis induction. The selective cytotoxicity and multitarget interaction profile of compound 4 highlight its potential as a promising lead compound for the development of novel therapeutic agents against breast cancer.

## 1 Introduction

Benzimidazole is a heterocyclic compound containing a benzene ring fused to an imidazole ring. Substitution with aliphatic groups (such as methyl, ethyl, or propyl) can modify the molecule's chemical properties, influencing its solubility, bioavailability, and overall stability (Pathare and Bansode 2021). Aliphatic substitutions often enhance the lipophilicity and pharmacokinetic properties, making them more suitable for drug development. These modifications can lead to compounds with improved cell membrane permeability (Choudhary et al. 2023). Aliphatic‐substituted benzimidazoles have been studied for their potential anticancer activities (Lee et al. 2023; Prasad and Kanvinde 2016). Some derivatives exhibit cytotoxic effects by inducing apoptosis, inhibiting cancer cell proliferation, and causing cell cycle arrest. These effects are commonly tested on various cancer cell lines, including breast, lung, and colon cancer cells (Atmaca et al. 2020; Ren et al. 2022; Nazreen et al. 2022). The anticancer activity may be attributed to their ability to interfere with essential cellular processes, such as DNA replication, repair, and apoptosis. Many benzimidazole derivatives inhibit tubulin polymerization, disrupt the mitotic spindle, and trigger cell death through apoptosis pathways (Ebenezer et al. 2023; Valdes‐García et al. 2022). In addition to anticancer activity, aliphatic‐substituted benzimidazoles have demonstrated antimicrobial and antiviral properties (Marinescu 2021, 2023). This makes them versatile candidates for the development of therapeutics against a wide range of infectious diseases.

Some derivatives act as enzyme inhibitors, targeting critical enzymes involved in cancer progression or microbial infections (Ibrahim and Refaat 2020; Hashem and El Bakri 2021). Recent studies have synthesized a variety of aliphatic‐substituted benzimidazole derivatives, which have shown promising results in preclinical trials (Bhambra et al. 2016; Carneiro and El‐Deiry 2020). Many of these studies focus on optimizing the pharmacokinetic properties and enhancing the specificity of these compounds for targeted therapies (Acar Çevik et al. 2023; Husain et al. 2021). While aliphatic‐substituted benzimidazoles show promise, their toxicity profiles need to be thoroughly evaluated (Lee et al. 2023). The modification of side chains can alter not only efficacy but also the safety profile, necessitating comprehensive toxicological assessments. Despite the encouraging in vitro results, many derivatives still require clinical trials to assess their therapeutic potential in humans fully.

## 2 Materials and Methods

### 2.1 Synthesis

For the structural characterization of the synthesized benzimidazole derivatives in this study, a Bruker AVANCE III 400 MHz NMR spectrometer, an Optimelt digital melting point apparatus, and an Agilent LC/MS‐TOF mass spectrometer were utilized.

General Procedure for Synthesis of 1. The synthesis of compound 1, which served as the starting material for our reactions, was carried out according to literature procedures, and its structure was confirmed.

#### 2.1.1 General Procedure for Synthesis of 2a

Compound **1** (0.01 mol) and phenyl isocyanate (0.01 mol) were refluxed in 40 mL of absolute ethanol in a 100 mL round‐bottom flask for 10 h. The reaction was monitored by TLC and completed. The resulting precipitate was collected by filtration and purified using ethanol. The product was dried under vacuum and identified as compound **2a**. Yield: 2.6 g (60%). M.p.: 232°C–233°C. ^1^H‐NMR (DMSO‐d_6_) ppm: 1.45, 1.65 (m, 6H, 2CH_3_), 3.48, 3.58 (m, 2H, CH_2_), 3.89, 4.06 (s, 2H, CH_2_), 6.10–6.16 (m, 3H, Ar‐H), 6.33–6.43 (m, 6H, Ar‐H), 6.49 (s, 1H, Ar‐H), 7.96 (s, 1H, NH), 8.63 (s, 1H, NH), 9.39 (s, 1H, NH), ^13^C‐NMR (DMSO‐d_6_) ppm: 20.31 (CH_3_), 20.54 (CH_3_), 28.17 (CH_2_), 44.88 (CH_2_), ArC: [110.62 (C), 119.30 (2C), 123.58 (C), 125.18 (C), 126.65 (C), 127.25 (2C), 129.16 (2C), 131.23 (C), 132.11 (C), 134.28 (C), 138.38 (C), 140.00 (C), 142.33 (C)], 153.28 (C = N), 155.13 (CH), 166.10 (C = O), 167.28 (C = O). LC/TOF‐MS, *m*/*z*: 434.1622 [M]^+^.

#### 2.1.2 General Procedure for Synthesis of 2b

Compound **1** (0.01 mol) and p‐chlorophenyl isocyanate (0.01 mol) were refluxed in 40 mL of absolute ethanol in a 100 mL round‐bottom flask for 10 h. The reaction was monitored by TLC and completed. The resulting precipitate was collected by filtration and purified using ethanol. The product was dried under vacuum and identified as compound **2b**. Yield: 3.98 g (85%). M.p.: 225°C–226°C. ^1^H‐NMR (DMSO‐d_6)_ ppm: 1.44, 1.65 (m, 6H, 2CH_3_), 3.47, 3.75 (s, 2H, CH_2_), 3.89, 4.25 (s, 2H, CH_2_), 6.10–6.13 (m, 3H, Ar‐H), 6.33 (s, 1H, Ar‐H), 6.48–6.54 (m, 4H, Ar‐H), 6.64 (s, 1H, Ar‐H), 7.80 (d, 1H, NH), 8.63 (s, 1H, NH), 9.39 (s, 1H, NH), ^13^C‐NMR (DMSO‐d_6_) ppm: 20.31 (CH_3_), 20.59 (CH_3_), 28.16 (CH_2_), 44.88 (CH_2_), ArC: [110.62 (C), 119.30 (2C), 125.59 (C), 126.72 (2C), 127.22 (2C), 129.00 (C), 130.18 (2C), 130.86 (2C), 134.55 (2C), 139.38 (C), 141.11 (C)], 152.76 (C = N), 166.45 (C = O), 174.80 (C = O). LC/TOF‐MS, *m*/*z*: 468.1220 [M]^+^.

#### 2.1.3 General Procedure for Synthesis of 3a

Compound **2a** (0.01 mol) in absolute ethanol was added to an aqueous solution of NaOH (0.01 mol). The resulting mixture was refluxed for 4.5 h. After completion of the reaction, the mixture was cooled to room temperature and poured into cold water. The pH of the solution was adjusted to 4 using dilute hydrochloric acid. The resulting mixture was kept in a refrigerator, and the precipitate that formed was collected and recrystallized from an alcohol–water mixture. The purified product was dried under vacuum and identified as compound **3a**. Yield: 2.8 g (65%); m.p.: 240°C–241°C. ^1^H‐NMR (DMSO‐d_6_) ppm: 1.44, 1.66 (m, 6H, 2CH3), 3.50, 3.59 (s, 2H, CH_2_), 3.90, 4.12 (s, 2H, CH_2_), 6.10, 6.17 (m, 3H, Ar‐H), 6.34, 644 (m, 6H, Ar‐H), 6.50‐6.54 (s, 1H, Ar‐H), 8.64,9.39 (s, 1H, OH), ^13^C‐NMR (DMSO‐d_6_) ppm: 20.30 (CH_3_), 20.54 (CH_3_), 28.15 (CH_2_), 44.79 (CH_2_), ArC: [110.79 (C), 119.29 (C), 122.51 (C), 125.63 (C), 126.72 (2C), 127.24 (2C), 129.14 (2C), 130.23 (C), 130.94 (C), 135.23 (C), 138.63 (C), 139.92 (C), 141.09 (C)], 152.69 (C = N), 156.13 (triazol C3), 167.22 (triazol C5). LC/TOF‐MS, *m*/*z*: 434.1651 [M]^+^.

#### 2.1.4 General Procedure for the Synthesis of 3b

Compound **2b** (0.01 mol) in absolute ethanol was added to an aqueous solution of NaOH (0.01 mol). The resulting mixture was refluxed for 6 h. After the reaction was completed, the mixture was cooled to room temperature and poured into ice‐cold water. The pH of the solution was adjusted to 4 using dilute hydrochloric acid. The mixture was then kept in a refrigerator, and the resulting precipitate was collected by filtration. The crude product was recrystallized from an alcohol–water mixture and dried under vacuum to afford compound **3b**. Yield: 2.51 g (55%); m.p.: 226°C–227°C. ^1^H‐NMR (DMSO‐d_6_) ppm: 1.43, 1.66 (s, 6H, 2CH_3_), 3.48, 3.58 (s, 2H, CH_2_), 3.89, 4.25 (s, 2H, CH_2_), 6.10–6.16 (m, 3H, Ar‐H), 6.42–6.55 (m, 5H, Ar‐H), 6.63 (s, 1H, Ar‐H), 8.63, 9.39 (s, 1H, OH), ^13^C‐NMR (DMSO‐d6) ppm: 20.30 (CH_3_), 20.58 (CH_3_), 28.16 (CH_2_), 44.88 (CH_2_), ArC: [110.78 (C), 119.30 (2C), 125.59 (C), 126.72 (2C), 127.25 (2C), 128.99 (C), 130.19 (2C), 130.87 (C), 134.63 (C), 138.13 (C), 139.36 (C), 141.53 (C)], 152.75 (C = N), 156.18 (triazol C3), 166.44 (triazol C5). LC/TOF‐MS, *m*/*z*: 434.1592 [M]^+^.

#### 2.1.5 General Procedure for the Synthesis of 4

Compound **2a** (0.01 mol) was stirred in 5 mL of 2 N H₂SO₄ solution at room temperature for 2 h. After completion, the reaction mixture was poured into cold water. The pH of the resulting solution was adjusted to the range of 5–6 using dilute aqueous ammonia. The mixture was then kept in the refrigerator for 12 h. The precipitate that formed was collected and recrystallized from an alcohol–water mixture. The purified product was dried under vacuum and identified as compound **4**. Yield: 2.7 g (65%); m.p.: 288°C–289°C. ^1^H‐NMR (DMSO‐d_6_) ppm: 1.47, 1.59 (s, 6H, 2CH_3_), 3.81, 3.87 (s, 2H, CH_2_), 4.43, 4.51 (s, 2H, CH_2_), 6.04, 6.17 (m, 3H, Ar‐H), 6.34 (d, J = 8.1 Hz, 1H, Ar‐H), 6.55 (m, 2H, Ar‐H), 6.67 (s, 2H, Ar‐H), 6.82 (s, 1H, Ar‐H), 7.42, 7.58 (s, 1H, Ar‐H), 9.53 (s, 1H, NH), ^13^C‐NMR (DMSO‐d_6_) ppm: 20.28 (CH_3_), 20.52 (CH_3_), 26.20 (CH_2_), 44.36 (CH_2_), ArC: [113.23 (C), 115.16 (C), 119.06 (C), 122.54 (C), 126.08 (2C), 127.87 (2C), 129.17 (2C), 129.33 (C), 131.54 (C), 134.18 (C), 136.15 (C), 137.23 (C), 151.11 (C)], 139.83 (oxadiazole C3), 151.73 (C = N), 165.10 (oxadiazole C5). LC/TOF‐MS, *m*/*z*: 417.2874 [M]^+^.

#### 2.1.6 General Procedure for Synthesis of 5a

Compound **1** (0.01 mol) and 4‐bromobenzaldehyde (0.01 mol) were refluxed in 30 mL of absolute ethanol in a 100 mL round‐bottom flask for 2 h. After completion of the reaction, the mixture was cooled, and the resulting precipitate was collected by filtration. The product was recrystallized from a 1:1 DMSO–water mixture and dried under vacuum. The obtained product was identified as compound **5a**. Yield: 3.4 g (72%). M.p.: 201°C–202°C. ^1^H‐NMR (DMSO‐d_6_) ppm: 1.43, 1.66 (m, 6H, 2CH_3_), 3.56, 3.59 (s, 2H, CH_2_), 4.11–4.56 (s, 2H, CH_2_), 6.08 (t, *J* = 2.3 Hz, 2H, Ar‐H), 6.37 (s, 1H, Ar‐H), 6.51 (m, 2H, Ar‐H), 6.81–6.89 (m, 4H, Ar‐H), 7.17, 7.37 (s, 1H, CH, A/Z amide conformer), 10.96, 11.04 (s, 1H, NH, E/Z amide conformer), ^13^C‐NMR (DMSO‐d_6_) ppm: 20.31 (CH_3_), 20.47 (CH_3_), 28.21 (CH_2_), 44.65 (CH_2_), ArC: [110.79 (C), 119.22 (C), 123.74 (C), 125.61 (C), 126.62 (C), 127.15 (C), 129.39 (2C), 130.06 (C), 130.84 (C), 132.28 (2C), 133.79 (C), 134.20 (C), 139.26 (C), 141.13 (C)], 143.33 (CH), 152.84 (C = N), 168.68 (C = O). LC/TOF‐MS, *m*/*z*: 483.0654 [M]^+^.

#### 2.1.7 General Procedure for Synthesis of 5b

Compound **1** (0.01 mol) and 4‐hydroxyphenyl isocyanate (0.01 mol) were refluxed in 30 mL of absolute ethanol in a 100 mL round‐bottom flask for 2 h. After completion of the reaction, the mixture was cooled, and the resulting precipitate was collected by filtration. The product was recrystallized from a 1:1 DMSO–water mixture and dried under vacuum. The obtained product was identified as compound **5b**. Yield: 2.8 g (68%). M.p.: 291°C–292°C. ^1^H‐NMR (DMSO‐d_6_) ppm: 1.43, 1.66 (m, 6H, 2CH_3_), 3.55‐3.59 (s, 2H, CH_2_), 4.07, 4.52 (s, 2H, CH_2_), 5.97 (d, *J* = 9.8 Hz, 2H, Ar‐H), 6.10 (d, *J* = 6.6 Hz, 2H, Ar‐H), 6.37 (s, 1H, Ar‐H), 6.52 (m, 2H, Ar‐H), 6.67–6.74 (m, 2H, Ar‐H), 7.09, 7.29 (s, 1H, CH, E/Z amide conformer), 9.11 (s, 1H, OH), 10.71, 10.78 (s, 1H, NH, E/Z amide conformer), ^13^C‐NMR (DMSO‐d_6_) ppm: 20.31 (CH_3_), 20.46 (CH_3_), 28.24 (CH_2_), 44.58 (CH_2_), ArC: [110.76 (C), 116.16 (2C), 119.21 (C), 125.59 (C), 126.61 (C), 127.15 (C), 127.80 (C), 128.20 (C), 129.24 (C), 129.42 (C), 131.40 (C), 134.96 (C), 139.30 (C), 141.14 (C), 159.83 (C)], 144.88 (CH), 152.85 (C = N), 168.18 (C = O). LC/TOF‐MS, *m*/*z*: 419.1524 [M]^+^.

#### 2.1.8 General Procedure for Synthesis of 5c

Compound **1** (0.01 mol) and 4‐methoxybenzaldehyde (0.01 mol) were refluxed in 30 mL of absolute ethanol in a 100 mL round‐bottom flask for 2 h. After completion of the reaction, the mixture was kept in a freezer, and the resulting precipitate was collected by filtration. The product was recrystallized from a 1:1 DMSO–water mixture and dried under vacuum. The obtained product was identified as compound **5c**. Yield: 2.3 g (55%). M.p.: 202°C–203°C. ^1^H‐NMR (DMSO‐d_6_) ppm: 1.43, 1.66 (m, 6H, 2CH_3_), 2.97 (d, J = 5.8 Hz, 3H, CH_3_), 3.56, 3.59 (s, 2H, CH_2_, E/Z amide conformer), 4.09, 4.53 (s, 2H, CH_2_, E/Z amide conformer), 6.13 (s, 2H, Ar‐H), 6.37 (d, *J* = 3.7 Hz, 2H, Ar‐H), 6.52 (s, 1H, Ar‐H), 6.83 (d, *J* = 8.7 Hz, 2H, Ar‐H), 7.14–7.33 (m, 3H, Ar‐H), 9.01 (s, 1H, CH), 10.78 (s, 1H, NH), ^13^C‐NMR (DMSO‐d_6_) ppm:: 20.33 (CH_3_), 20.57 (CH_3_), 28.22 (CH_2_), 44.60 (CH_2_), 55.78 (CH_3_), ArC: [110.79 (C), 114.80 (C), 119.20 (C), 125.63 (C), 126.63 (C), 127.04 (2C), 127.16 (2C), 129.10 (C), 130.04 (C), 130.84 (C), 135.04 (C), 139.18 (C), 141.10 (C), 144.18 (C)], 152.85 (C = N), 161.39 (CH), 168.33 (C = O). LC/TOF‐MS, *m*/*z*: 433.1681 [M]^+^.

#### 2.1.9 General Procedure for Synthesis of 6

Compound **1** (0.01 mol) in a 100 mL round‐bottom flask, a mixture of CS₂ (0.001 mol) and KOH (0.001 mol) in 60 mL of water was added. The mixture was refluxed for 4 h. After cooling to room temperature, the reaction mixture was neutralized with HCl, and the resulting precipitate was collected by filtration and recrystallized from an ethanol–water mixture. The purified compound was identified as compound **6**. Yield: 2.92 g (82%). M.p.: 245°C–246°C. ^1^H‐NMR (DMSO‐d_6_) ppm: 1.44, 1.64 (m, 6H, 2CH_3_), 3.68 (s, 2H, CH_2_), 4.81 (s, 2H, CH_2_), 6.05–6.08 (m, 2H, Ar‐H), 6.51–6.54 (m, 3H, Ar‐H), 13.50 (bs, 1H, SH), ^13^C‐NMR (DMSO‐d_6_) ppm: 20.29 (CH_3_), 20.60 (CH_3_), 27.83 (CH_2_), 38.63 (CH_2_), ArC: [110.88 (C), 119.28 (C), 125.84 (C), 126.81 (C), 127.12 (C), 131.18 (C), 131.83 (C), 134.00 (C), 138.75 (C), 140.47 (C)], 152.32 (C = N), 159.53 (oxadiazole C3), 178.35 (oxadiazole C5). LC/TOF‐MS, m/z: 357.0826 [M]^+^.

### 2.2 Cell Culture

Human breast cancer cell lines MCF‐7 and MDA‐MB‐231 were obtained from Leibniz Institute DSMZ (Germany). Human normal breast epithelial cells MCF‐10A were obtained from the American Type Culture Collection ATCC (USA). Cancer cell lines were cultured in RPMI 1640 medium supplemented with 10% heat‐inactivated fetal bovine serum, 100 μg/mL streptomycin, and 100 U/mL penicillin. MCF‐10A cells were maintained in DMEM/F12 (Dulbecco′s Modified Eagle Medium/Nutrient Mixture F‐12) medium with 5% horse serum, 20 ng/mL epidermal growth factor (EGF), 0.5 µg/mL hydrocortisone, 100 ng/mL cholera toxin, 10 µg/mL insulin, and 1% penicillin‐streptomycin (100 U/mL penicillin, 100 μg/mL streptomycin). The cells were incubated in a humidified incubator at 37°C with 5% CO_2_. The viability, proliferation, and infection status of the cells were monitored daily using an inverted microscope. Passaging was performed when the cell density reached over 80%, and the cells were stored at −80°C.

### 2.3 Cell Viability Assay

The synthesized compounds were dissolved in DMSO to prepare stock solutions. The effects of the compounds on cell viability were determined using the MTT assay (3‐(4,5‐dimethylthiazol‐2,5‐diphenyltetrazolium bromide). The MTT assay is based on the reduction of MTT by mitochondrial dehydrogenase enzymes in living cells to form formazan crystals. These crystals are then dissolved in dimethyl sulfoxide (DMSO), and the optical density is measured spectrophotometrically. The amount of color produced is considered directly proportional to cell viability. To assess the effects of the compounds on the viability of breast cancer cells, 10^4^ cells per well were seeded into 96‐well plates. The plates were incubated at 37°C for 24 h to allow the cells to adhere. At the end of the incubation period, dilutions of the compounds were added to the wells. After the treatment incubation, 20 μL of MTT solution was added to each well. The plates with MTT were incubated at 37°C for 4 h. Following incubation, the solution in each well was removed, and 200 μL of DMSO was added to dissolve the formazan crystals. The plates were shaken for 5 min to allow color development, and absorbance was measured at 570 nm using a microplate reader (Atmaca et al. 2020).

### 2.4 Analysis of Cell Cycle Distribution Using Flow Cytometry

To evaluate the cell cycle distribution, DNA fragmentation was assessed using propidium iodide (PI) staining, with analysis conducted using the Cell Cycle Phase Determination Kit (Cayman Chemical, USA). Cells were seeded in six‐well plates at a density of 1 × 10⁶ cells per well in 2 mL of culture medium, followed by incubation at 37°C in a CO₂ incubator for 72 h. After this period, the cells were treated with the IC_50_ concentrations of compound **4** for an additional 72 h. After treatment, cells were collected by centrifugation, washed twice with cold phosphate‐buffered saline (PBS), and then fixed by adding 1 mL of fixative. The fixation step lasted for 2 h, followed by a second centrifugation to remove the fixative. The cells were then resuspended in 0.5 mL of a staining solution containing 200 µL of DNase‐free RNase (Sigma–Aldrich) and PI. The suspension was incubated for 30 min at room temperature and protected from light. Finally, the cell cycle distribution was determined using an Accuri C6 flow cytometer (BD Biosciences, USA). This technique allowed for the analysis of cell cycle progression and its alterations induced by the treatment (Ilhan et al. 2023a).

### 2.5 Apoptosis Detection via Annexin V and Propidium Iodide Staining

Annexin V, a protein that binds strongly to phosphatidylserine (PS), is commonly used in apoptosis detection. PS is normally located on the inner side of the plasma membrane, but during apoptosis, it translocates to the outer surface, making it a key indicator of apoptotic cells (Atmaca et al. 2020). Annexin V specifically binds to PS exposed on the outer leaflet of apoptotic cells. To distinguish between apoptotic and viable cells, propidium iodide (PI), a secondary dye, is used. This allows for the identification of live cells (Annexin V‐, PI‐) and apoptotic cells (Annexin V + , PI + ). In this study, the FITC Annexin V Apoptosis Detection Kit I (BD Pharmingen) was used for apoptosis analysis. Cancer cells were seeded in 6‐well plates at a density of 1 × 10⁶ cells per well and treated with the IC_50_ concentration of **4** for 72 h. After treatment, the cells were washed with cold PBS, resuspended in 1 mL of 1X Binding Buffer, and stained with 5 µL of Annexin V FITC and 5 µL of PI. The samples were vortexed and incubated in the dark at room temperature (25°C) for 15 min. Following incubation, 400 µL of 1X Binding Buffer was added, and apoptosis was assessed using the BD Accuri C6 Flow Cytometer (Ilhan et al. 2023b). This method allows for the precise identification and analysis of apoptotic cells within the treated cell population.

### 2.6 Molecular Docking and MM‐GBSA Binding Free Energy Calculations of Compound 4

Molecular docking simulations were conducted to examine the interactions of compound **4** with proteins associated with apoptosis regulation and cell cycle arrest, particularly in the S phase. Autodock Vina (version 4.2.5.1) was employed for these simulations. The 3D structures of the target proteins, which include Bcl‐2 (PDB ID: 2W3L), Bcl‐xL (PDB ID: 2YXJ), CDK2 (PDB ID: 2VU3), and Cyclin B1 (PDB ID: 2B9R), were retrieved from the RCSB Protein Data Bank (https://www.rcsb.org/). These protein structures were prepared using the Protein Preparation Wizard, which added hydrogen atoms, adjusted bond orders, and processed metal ions, while removing water molecules. Energy minimization of the proteins was performed, achieving a root mean square deviation (RMSD) of 0.30 Å for optimal docking conditions.

The 3D structure of compound **4** was generated using Maestro 8.5 (Schrödinger), and Open Babel was used for the other synthesized compounds. The docking grid parameters were set to achieve RMSD values below 2 Å, with the grid center coordinates for proteins placed at *X* = 21.41, *Y* = 3.62, and *Z* = 21.94, and the grid dimensions set to 60 × 60 × 60 Å. These settings were consistent across all proteins after calibration.

Docking results provided multiple binding conformations, which were analyzed using Discovery Studio for secondary structure exploration. Cisplatin (CP) served as a reference compound in the study. For the ligand preparation, Gaussian 09 W was used to optimize the chemical structure of compound **4**, and the optimized structure was saved in SDF format. This structure was then imported into the Maestro graphical user interface (GUI), where ligand preparation was performed with default parameters using the OPLS 2005 force field at physiological pH. After preparation, the ligands were docked into the receptor grid, which had a radius of 20 Å. Co‐crystallized ligands were employed to confirm the active binding site, and the redocking process resulted in RMSD values below 2 Å, validating the docking method′s accuracy and reliability (Atmaca et al. 2023).

MM‐GBSA binding free energy calculations were performed to further refine the molecular docking results and evaluate the energetic stability of compound 4 within the selected protein targets. The docked protein–ligand complexes obtained from AutoDock Vina were used as input structures for the MM‐GBSA analysis. Binding free energies (ΔG_bind) were calculated by combining van der Waals (ΔE_vdW), electrostatic (ΔE_ele), and solvation energy (ΔG_solv) contributions. All MM‐GBSA calculations were carried out using standard force‐field parameters under implicit solvent conditions. The reported binding free energy values represent averaged estimates derived from the minimized docked complexes. MM‐GBSA calculations were performed using widely adopted protocols implemented in commonly used molecular modeling packages.

### 2.7 ADMET Analysis

The pharmacokinetic and toxicity‐related properties of compound 4 were evaluated using the AdmetSAR 2.0 web‐based prediction server (http://lmmd.ecust.edu.cn/admetsar2). This computational tool was employed to estimate key absorption, distribution, metabolism, excretion, and toxicity (ADMET) parameters, providing an in silico assessment of the compound′s drug‐likeness profile. The analysis generated predictions for multiple physicochemical descriptors, including molecular weight, octanol/water partition coefficient (LogP), aqueous solubility (log S), skin permeability (log Kp), hydrogen bond donors and acceptors, topological polar surface area, and molar refractivity. Together, these parameters offer a comprehensive overview of the compound′s potential behavior in biological systems. To evaluate suitability as a drug candidate, the predicted properties were interpreted in the context of Lipinski′s Rule of Five and commonly accepted ADME criteria. According to these guidelines, favorable drug‐like characteristics include a molecular weight not exceeding 500 Da, no more than 10 hydrogen bond acceptors and 5 hydrogen bond donors, a LogP value ≤ 5, and a molar refractivity ≤ 140. In addition, acceptable ranges for oral drug candidates include LogP values between −2 and 6.5, polar surface area between 7 and 200 Å², aqueous solubility values above −4 (log S), and a predicted drug score greater than 0.5. These criteria were used as benchmarks to assess the overall pharmacokinetic suitability of compound 4.

## 3 Results

### 3.1 Compound 4 Demonstrates Potent Cytotoxic Effect in Breast Cancer Cell Lines

The cytotoxic effects of the synthesized compounds were evaluated in MCF‐7 and MDA‐MB‐231 breast cancer cell lines, as well as in the non‐tumorigenic breast epithelial cell line MCF‐10A, using the MTT assay after 72 h of treatment. The half‐maximal inhibitory concentration (IC₅₀) values of each compound are summarized in Table 1.

**Table 1:** IC₅₀ values (µM) of the synthesized compounds in MCF‐7 and MDA‐MB‐231 breast cancer cell lines and MCF‐10A non‐tumorigenic breast cells after 72 h of treatment.

	IC_50_ (µM)		
Compound	MCF‐7	MDA‐MB‐231	MCF‐10A
2a	45.4 ± 0.9	82.3 ± 1.5	64.7 ± 1.3
2b	154.3 ± 3.1	114.9 ± 2.0	162.7 ± 3.0
3a	18.7 ± 1.6	20.4 ± 2.0	14.4 ± 0.8
3b	52.8 ± 0.8	43.3 ± 1.8	44.7 ± 2.4
4	12.8 ± 1.0	15.7 ± 2.1	16.4 ± 2.0
5a	91.4 ± 1.6	100.2 ± 3.1	117.6 ± 2.2
5b	115.3 ± 1.7	108.6 ± 1.2	121.9 ± 2.0
5c	59.1 ± 1.3	66.5 ± 1.1	50.8 ± 1.4
6	116.3 ± 2.7	120.0 ± 1.9	125.7 ± 2.3
Cisplatin	14.7 ± 0.8	19.9 ± 0.6	15.4 ± 1.2

Among the tested compounds, compound **4** exhibited the strongest cytotoxic activity against both MCF‐7 and MDA‐MB‐231 cells, with IC₅₀ values of 12.8 ± 1.0 µM and 15.7 ± 2.1 µM, respectively. These values were comparable to those of the reference drug cisplatin (IC₅₀ = 14.7 ± 0.8 µM for MCF‐7 and 19.9 ± 0.6 µM for MDA‐MB‐231). In contrast, compound 3a also demonstrated notable cytotoxicity, with IC₅₀ values of 18.7 ± 1.6 µM and 20.4 ± 2.0 µM in MCF‐7 and MDA‐MB‐231 cells, respectively. Compounds 2a, 3b, 5a, 5b, 5c, and 6 showed varying degrees of activity, with IC₅₀ values ranging from moderate to weak when compared to cisplatin. For example, compound 2a displayed an IC₅₀ of 45.4 ± 0.9 µM in MCF‐7 and 82.3 ± 1.5 µM in MDA‐MB‐231 cells, indicating a relatively lower potency. Similarly, compounds 5a–5c and 6 exhibited IC₅₀ values above 59 µM, suggesting less pronounced cytotoxic effects at 72 h. Evaluation in non‐tumorigenic MCF‐10A cells revealed that most compounds exhibited higher IC₅₀ values compared to cancer cell lines, indicating a certain degree of selectivity. In particular, compound **4** showed an IC₅₀ of 16.4 ± 2.0 µM in MCF‐10A cells, suggesting a favorable therapeutic window.

Overall, the MTT assay results indicated that compound **4** possessed the highest cytotoxic potential among the synthesized molecules, with a potency comparable to cisplatin and a moderate selectivity toward cancer cells. These findings highlight compound **4** as a promising candidate for further investigation in anticancer studies.

### 3.2 Compound 4 Induces S‐Phase Cell Cycle Arrest in MCF‐7 and MDA‐MB‐231 Cells

**Figure 1:**
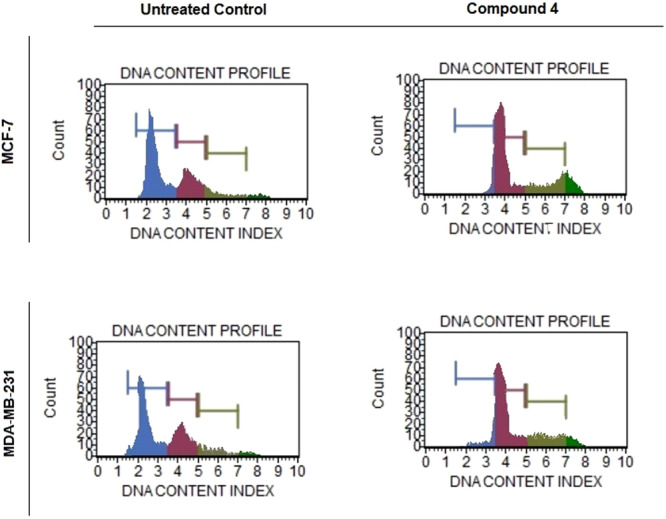
Cell cycle distribution of MCF‐7 and MDA‐MB‐231 cells after treatment with compound 4. Cells were exposed to the IC₅₀ concentration of compound 4 for 72 h and analyzed using propidium iodide (PI) staining by flow cytometry. Histograms indicate the percentage of cells in G₀/G₁, S, and G₂/M phases in control and treated groups. Compound 4 induced a marked accumulation of cells in the S phase, indicating inhibition of DNA synthesis and replication stress–mediated cell cycle arrest.

To investigate whether compound **4** affects cell cycle progression, MCF‐7 and MDA‐MB‐231 breast cancer cells were treated with its IC₅₀ concentration for 72 h and analyzed using propidium iodide (PI) staining and flow cytometry. Representative DNA content histograms are presented in Figure 1.

In the untreated control groups, both cell lines displayed a typical proliferative profile, with the majority of cells in the G0/G1 phase and smaller populations in the S and G2/M phases. Specifically, MCF‐7 control cells showed approximately 55.6% in G0/G1, 31.5% in S, and 9.9% in G2/M phases, while MDA‐MB‐231 control cells exhibited 58.1% in G0/G1, 30.7% in S, and 9.2% in G2/M phases.

After 72 h of treatment with compound **4**, both MCF‐7 and MDA‐MB‐231 cells demonstrated marked alterations in their cell cycle distribution. In MCF‐7 cells, the proportion of cells in the G0/G1 phase decreased to approximately 38%–40%, accompanied by an increase in the S phase population (45%–47%) and a moderate rise in G2/M phase cells (12‐14%). Similarly, MDA‐MB‐231 cells showed a reduction in the G0/G1 fraction to around 40%–42%, with concomitant accumulation in the S (43%–45%) and G2/M (13%–15%) phases.

These findings indicate that compound **4** induces a cell cycle arrest predominantly at the S phase, suggesting an interference with DNA synthesis and replication processes. The observed accumulation of cells in S and G2/M phases is consistent with a mechanism involving replication stress or checkpoint activation, contributing to the antiproliferative activity of compound **4**.

### 3.3 Compound 4 Promotes Apoptotic Cell Death in Breast Cancer Cells

**Figure 2:**
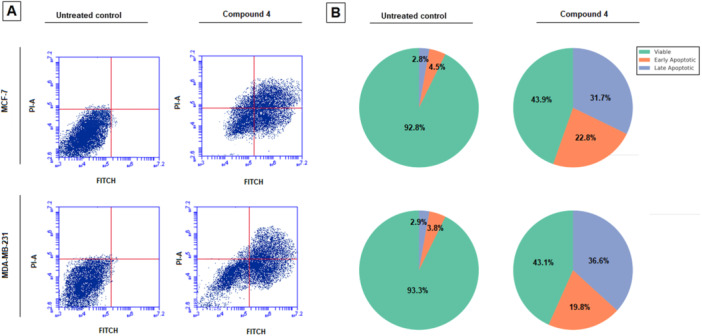
Effect of Compound 4 on apoptotic cell distribution in breast cancer cells. (A) MCF‐7 and MDA‐MB‐231 breast cancer cells were treated with the IC₅₀ concentration of compound 4 for 72 h, and apoptosis was analyzed by FITC Annexin V/PI double staining followed by flow cytometry. (B) Pie charts represent the percentage of viable, early apoptotic, and late apoptotic/necrotic cells in control and treated groups. A marked increase in apoptotic cell populations was observed after treatment, consistent with the cytotoxic and cell cycle arrest effects of compound 4.

To determine whether the cytotoxic effect of compound **4** was associated with the induction of apoptosis, MCF‐7 and MDA‐MB‐231 breast cancer cells were treated with the IC₅₀ concentration of the compound for 72 h and analyzed using the Annexin V/PI double‐staining method. The distribution of viable, early apoptotic, late apoptotic, and necrotic cells was quantified by flow cytometry (Figure 2).

In the untreated control groups, the majority of cells remained viable, with MCF‐7 showing 92.8% viable, 4.5% early apoptotic, and 2.8% late apoptotic/necrotic populations, while MDA‐MB‐231 cells exhibited 93.3% viable, 3.8% early apoptotic, and 2.9% late apoptotic/necrotic populations. Following 72 h treatment with compound **4**, a marked increase in apoptotic cell populations was observed in both cell lines. In MCF‐7 cells, the percentage of early apoptotic cells increased to 22.8%, while late apoptotic/necrotic cells reached 31.7%, reducing the viable cell fraction to approximately 43.9%. Similarly, MDA‐MB‐231 cells exhibited 19.8% early apoptosis and 36.6% late apoptosis/necrosis, with viable cells decreasing to 43.1%.

These results indicate that compound **4** effectively induces apoptosis in both hormone receptor–positive (MCF‐7) and triple‐negative (MDA‐MB‐231) breast cancer cells. The significant increase in Annexin V–positive populations suggests that apoptosis is a major mechanism underlying the cytotoxic action of compound **4**, consistent with its observed cell cycle arrest and antiproliferative effects.

### 3.4 Molecular Docking and MM‐GBSA Binding Free Energy Calculations

Molecular docking studies were performed to investigate the potential interaction of the synthesized compound 4 with key regulatory proteins involved in apoptosis and cell cycle control, including Bcl‐2, Bcl‐xL, CDK2, and Cyclin E. The binding energies and hydrogen bond interactions are summarized in Table 2. Compound 4 exhibited strong binding affinities with all four target proteins, suggesting a multitarget mechanism of action that may contribute to its observed cytotoxic and pro‐apoptotic effects in breast cancer cells. Among these, the CDK2 complex demonstrated the lowest binding energy (–164.055 kcal/mol), indicating the most stable interaction, followed closely by Bcl‐xL (–158.828 kcal/mol). The interactions with Cyclin E (–140.654 kcal/mol) and Bcl‐2 (–127.644 kcal/mol) were also energetically favorable. For CDK2, compound 4 formed hydrogen bonds with multiple residues, including LEU134, ASP145, GLN131, and LYS33, located near the ATP‐binding pocket. These interactions likely interfere with kinase activity and contribute to the cell cycle arrest observed in the biological assays.

**Table 2:** Molecular docking analysis of compound 4 with Bcl‐2, Bcl‐xL, CDK2, and Cyclin E proteins.

Protein name	Docking score (Binding Energy, Kcal/mol)	H Bond	Amino acid residue
Bcl‐2	−127.644	−13.688	ARG26, ARG65, ARG68, PHE71, VAL118, GLU119, VAL115, VAL118, LYS22, SER64, ASP61
Bcl‐xL	−158.828	−0.641	TYR101, TYR195, PHE97, ARG100, GLU92, GLU96, ASN136, GLY138, ALA93, ALA142, ARG139, VAL141, TRP137
CDK2	−164.055	−0.349	LEU134, VAL64, ALA144, ASP145, ASN132, GLN131, LYS33, LYS129, TYR15, TYR159, GLU12, GLU162, GLY13, GLY16, THR14, ILE35, VAL18, PHE80
Cyclin E	−140,654	−3.759	MET105, GLU149, SER233, VAL101, VAL237, VAL250, TRP95, PRO91, LEU90, LEU93, LEU251, ASN236, TYR255, GLN240, PRO253

**Figure 3:**
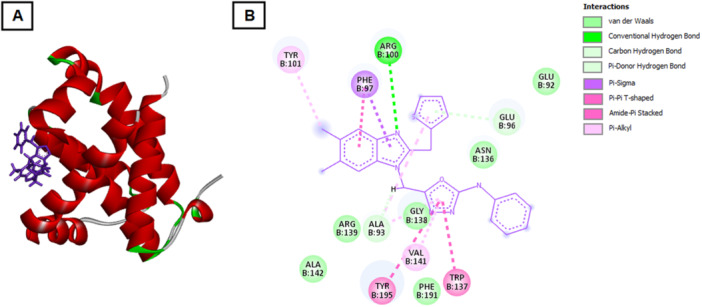
Molecular docking interaction of compound 4 with the Bcl‐xL protein. (A) Three‐dimensional binding pose of compound 4 within the hydrophobic pocket of Bcl‐xL. The compound interacts with key residues TYR101, PHE97, ARG100, and TRP137, stabilizing the complex. (B) Two‐dimensional interaction map showing hydrogen bonds and hydrophobic contacts between compound 4 and Bcl‐xL residues, indicating potential inhibition of Bcl‐xL′s anti‐apoptotic function.

**Figure 4:**
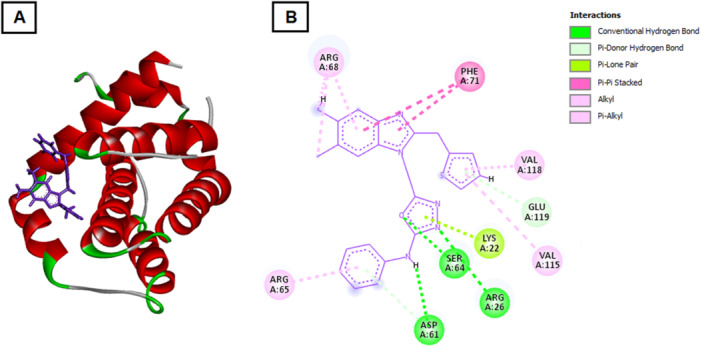
Docking pose of compound 4 in the Bcl‐2 binding pocket. (A) 3D docking representation illustrating hydrogen bonding of compound 4 with ARG26, ARG65, and GLU119 residues in the Bcl‐2 active region. (B) 2D interaction diagram highlighting hydrogen bonds and hydrophobic contacts that may disrupt Bcl‐2′s association with Bax/Bak, promoting apoptotic signaling.

In the Bcl‐xL complex, several key residues such as TYR101, PHE97, ARG100, and TRP137 participated in hydrogen bonding and hydrophobic interactions, stabilizing the ligand within the hydrophobic groove responsible for sequestering pro‐apoptotic proteins (Figure 3). This suggests that compound 4 may act as a Bcl‐xL inhibitor, promoting mitochondrial‐mediated apoptosis. Similarly, interaction with Bcl‐2 involved hydrogen bonding with ARG26, ARG65, and GLU119, residues critical for the anti‐apoptotic function of the protein (Figure 4). Such binding may prevent Bcl‐2 from interacting with Bax/Bak, thereby triggering apoptotic cascades.

**Figure 5:**
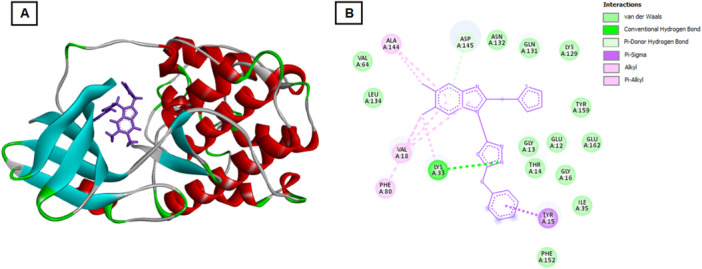
Binding interaction of compound 4 with CDK2. (A) 3D view showing compound 4 occupying the ATP‐binding cleft of CDK2. Hydrogen bonds are observed with LEU134, ASP145, GLN131, LYS33, and TYR15 residues. (B) 2D schematic representation of hydrogen bonding and hydrophobic interactions (VAL64, ILE35, VAL18, PHE80), suggesting inhibition of ATP binding and kinase activity.

Compound 4 demonstrated the strongest binding affinity toward CDK2 (–164.055 kcal/mol), indicating a particularly stable interaction compared to the other targets analyzed. The molecule engaged in hydrogen bonding with key active‐site residues such as LEU134, ASP145, GLN131, LYS33, and TYR15, which are positioned within or adjacent to the ATP‐binding pocket of CDK2 (Figure 5). These residues are known to play critical roles in stabilizing ATP and coordinating phosphorylation events essential for CDK2 kinase activity. Additionally, hydrophobic interactions with residues like VAL64, ILE35, VAL18, and PHE80 further strengthened the ligand′s accommodation within the catalytic cleft. Such interactions can prevent ATP binding and inhibit substrate phosphorylation, effectively suppressing CDK2 enzymatic function.

**Figure 6:**
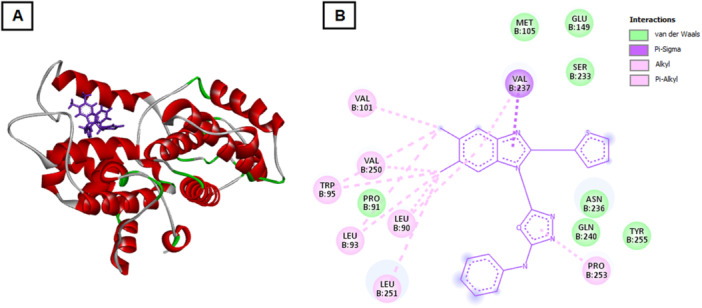
Docking conformation of compound 4 with Cyclin E. (A) 3D docking pose displaying the interaction of compound 4 with residues GLU149, SER233, and TYR255 near the Cyclin E–CDK2 interface. (B) 2D interaction diagram illustrating hydrogen bonds and hydrophobic contacts that may destabilize the Cyclin E/CDK2 complex, supporting the observed G1/S phase arrest.

Docking with Cyclin E revealed interactions with residues GLU149, SER233, and TYR255, positioned at the Cyclin E‐CDK2 interface (Figure 6). This may destabilize the Cyclin E/CDK2 complex formation, further supporting the compound′s potential to induce G1/S phase arrest.

Overall, the docking data indicate that compound 4 exhibits high affinity toward both apoptotic (Bcl‐2, Bcl‐xL) and cell cycle regulatory (CDK2, Cyclin E) targets. These results are consistent with the in vitro findings, supporting the hypothesis that the compound exerts its anticancer activity through a dual mechanism involving apoptosis induction and cell cycle inhibition.

MM‐GBSA binding free energy calculations were performed to refine docking predictions and evaluate the energetic stability of compound 4 within the selected targets. As shown in Table X, compound 4 exhibited favorable binding free energies toward all four proteins, with the strongest affinity observed for CDK2 (ΔG_bind = −48.3 kcal/mol) and Bcl‐2 (ΔG_bind = −45.1 kcal/mol). The binding energetics were predominantly driven by van der Waals interactions, consistent with the presence of an extended aliphatic substituent that efficiently occupies hydrophobic pockets. These findings further support the proposed dual mechanism of action involving apoptosis induction and cell‐cycle arrest.

### 3.5 ADMET Analysis of Compound 4

The ADMET properties of compound 4 were evaluated using in silico prediction tools to assess its physicochemical characteristics and drug‐likeness. According to Lipinski′s rule of five (Ro5), orally active drug candidates are expected to present no more than one violation. In the present analysis, compound 4 complied fully with Ro5 criteria. The molecular weight of compound 4 was below 500 Da, while the numbers of hydrogen bond acceptors and donors were within the acceptable limits, indicating favorable drug‐like properties. Lipophilicity, expressed as the logarithm of the n‐octanol/water partition coefficient (LogP), plays a crucial role in membrane permeability and absorption. Compound 4 exhibited a moderate LogP value, suggesting an optimal balance between lipophilicity and aqueous solubility. The total polar surface area (TPSA), which reflects the contribution of polar atoms such as oxygen and nitrogen, was within the recommended threshold (<140 Å²), indicating a high likelihood of membrane permeability. Based on TPSA values, the predicted percentage of absorption (%ABS) for compound 4 was high, supporting efficient cellular uptake.

In addition, compound 4 demonstrated acceptable aqueous solubility, with a predicted logS value indicative of moderate solubility in water. Human intestinal absorption predictions suggested favorable oral absorption, while the predicted bioavailability scores (20% and 30%) exceeded the recommended thresholds, indicating good systemic exposure potential. These findings collectively suggest that compound 4 possesses a favorable absorption and bioavailability profile. Metabolic liability predictions indicated that compound 4 is unlikely to cause significant cytochrome P450 inhibition, reducing the risk of drug–drug interactions. Distribution‐related parameters suggested moderate plasma protein binding and low blood–brain barrier permeability, which may be advantageous for minimizing central nervous system–related side effects in anticancer therapy. Furthermore, toxicity predictions revealed a negative Ames mutagenicity result and a low risk of hERG channel inhibition, indicating a favorable preliminary safety profile. The predicted rat oral toxicity value suggested low acute oral toxicity.

Overall, the in silico ADMET analysis indicates that compound 4 exhibits suitable physicochemical properties, favorable pharmacokinetic behavior, and low predicted toxicity, supporting its potential as a promising lead compound for further anticancer drug development. Detailed ADMET prediction results are provided in Table S1.

## 4 Discussion

The present study investigated a new series of aliphatic‐substituted benzimidazole derivatives and demonstrated that structural modifications at the benzimidazole core markedly influence their biological activity. Among the synthesized molecules, compound 4 displayed the most pronounced cytotoxic and pro‐apoptotic effects against MCF‐7 and MDA‐MB‐231 breast cancer cell lines, while showing limited toxicity in normal MCF‐10A cells. This selectivity highlights its potential as a lead compound for the development of safer and more effective anticancer agents compared to traditional cytotoxic drugs such as cisplatin.

The superior activity of compound 4 can be rationalized by its unique chemical architecture. The presence of two aliphatic methyl groups and a heteroaromatic oxadiazole ring likely enhances both lipophilicity and electronic conjugation, facilitating cellular uptake and interaction with intracellular targets (Srour et al. 2020; El‐Gohary and Shaaban 2017). Previous reports have emphasized that benzimidazole scaffolds substituted with electron‐donating or moderately hydrophobic groups display improved cell permeability and affinity toward biological macromolecules (Guo et al. 2020). The proton‐donor ability of the NH group, combined with π–π stacking interactions from the fused aromatic system, provides a favorable balance between hydrophilic and lipophilic characteristics, which may contribute to the observed bioactivity profile (Cichero et al. 2021).

The cytotoxicity data indicated that compound 4 inhibits cell viability in a concentration‐dependent manner, producing IC₅₀ values close to those of cisplatin. Flow‐cytometric analyses revealed that this growth suppression is associated with a pronounced S‐phase arrest, implying an interference with DNA synthesis or replication checkpoints. Arrest at this phase prevents cells from completing DNA duplication, leading to the activation of damage‐response pathways and subsequent apoptotic signaling (Lee et al. 2023). Such behavior is consistent with the ability of benzimidazole derivatives to interact with DNA and enzymes regulating replication fidelity.

Apoptosis assays supported this mechanism, showing a significant increase in both early and late apoptotic populations following treatment with compound 4. The simultaneous S‐phase accumulation and apoptotic shift suggest that the compound activates programmed cell death following replication stress. Literature reports support that benzimidazole analogues can induce mitochondrial‐dependent apoptosis through elevated reactive oxygen species (ROS) production and dissipation of the mitochondrial membrane potential (Ren et al. 2022; Yang et al. 2014). Although ROS levels were not directly measured here, the pattern of Annexin V positivity and reduced viable cell counts imply the engagement of mitochondrial pathways.

To gain insight into the molecular basis of these biological outcomes, molecular docking analyses were performed against proteins implicated in apoptosis and cell cycle control: Bcl‐2, Bcl‐xL, CDK2, and Cyclin B1 (Youssif et al. 2024). The docking simulations revealed that compound 4 formed stable complexes with binding energies comparable to or stronger than those of cisplatin. In particular, interactions with Bcl‐2 and Bcl‐xL occurred through hydrogen bonding with critical residues within the BH3‐binding groove, which may inhibit the anti‐apoptotic function of these proteins and promote mitochondrial outer‐membrane permeabilization. This mechanistic inference aligns with the observed increase in apoptotic cell populations.

To further validate the docking results, MM‐GBSA binding free energy calculations were performed to refine docking predictions and evaluate the energetic stability of compound 4 within the selected targets. As shown in Table X, compound 4 exhibited favorable binding free energies toward all four proteins, with the strongest affinity observed for CDK2 (ΔG_bind = −48.3 kcal/mol) and Bcl‐2 (ΔG_bind = −45.1 kcal/mol). The binding energetics were predominantly driven by van der Waals interactions, consistent with the presence of an extended aliphatic substituent that efficiently occupies hydrophobic pockets. These findings further support the proposed dual mechanism of action involving apoptosis induction and cell‐cycle arrest. These calculations provide energetic validation of the docking poses without the need for extended molecular dynamics simulations.

Although the benzimidazole scaffold has been extensively explored in anticancer drug discovery, recent studies continue to demonstrate that rational structural modification can yield derivatives with pronounced antiproliferative and mechanistic effects. For instance, Syed Nazreen et al. reported a series of benzimidazole derivatives capable of inducing cell cycle arrest and apoptosis in cancer cells, supported by molecular docking analyses that highlighted favorable interactions with apoptosis‐ and cell‐cycle‐related targets (Nazreen et al. 2022). Their findings emphasize that appropriately substituted benzimidazoles can simultaneously modulate cell proliferation and programmed cell death pathways, in line with the biological profile observed for compound 4 in the present study. In another relevant work, Almalki et al. designed and synthesized benzimidazole derivatives bearing 1,3,4‐oxadiazole moieties and demonstrated significant anticancer activity through inhibition of thymidylate synthase (Almalki et al. 2022). Molecular docking studies revealed stable binding modes driven by hydrophobic and hydrogen‐bonding interactions, underscoring the importance of oxadiazole incorporation in enhancing target affinity and biological performance. Notably, the presence of an oxadiazole fragment in compound 4 similarly appears to contribute to its strong binding energetics and multitarget behavior, as supported by docking and MM‐GBSA analyses. Furthermore, Alzahrani et al. reported benzimidazole derivatives acting as EGFR inhibitors, exhibiting potent antiproliferative effects supported by docking and DFT studies (Alzahrani et al. 2022). Their work highlights that substitution patterns around the benzimidazole core critically influence electronic distribution, binding orientation, and biological potency. Together, these recent studies corroborate that while the benzimidazole nucleus is a well‐established pharmacophore, strategic functionalization‐particularly with heterocyclic and lipophilic substituents‐remains an effective approach to achieve enhanced anticancer activity. In this context, the aliphatic substitution and oxadiazole hybridization employed in compound 4 represent a rational extension of current benzimidazole‐based anticancer design strategies.

In addition to its favorable biological and binding profiles, compound 4 exhibited a promising in silico ADMET profile. The predicted high gastrointestinal absorption and moderate lipophilicity suggest favorable oral bioavailability, while the absence of significant cytochrome P450 inhibition indicates a reduced risk of drug‐drug interactions. Moreover, the low predicted blood–brain barrier permeability may be advantageous in minimizing central nervous system–related adverse effects. Toxicity‐related predictions further supported the drug‐like nature of compound 4, showing a negative Ames mutagenicity outcome and a low risk of hERG channel inhibition, together with low predicted acute oral toxicity. Although these results are based on computational estimations, they provide supportive evidence for the further development of compound 4 as a lead anticancer candidate (Table S1).

Furthermore, the binding orientation of compound 4 within CDK2 and Cyclin B1 active sites indicated potential interference with kinase activity responsible for S‐phase progression and the G₂/M transition. The compound engaged key amino acids within the ATP‐binding cleft via π–π stacking and hydrophobic interactions, stabilizing the inactive conformation of the complex. These findings provide a molecular rationale for the S‐phase accumulation detected by flow cytometry, suggesting that cell‐cycle arrest and apoptosis induction may arise from simultaneous modulation of kinase‐dependent signaling and mitochondrial regulation (El‐Hameed et al. 2021).

The structural features that enable compound 4 to interact with both apoptotic and cell‐cycle regulatory proteins illustrate its multitarget profile, which is highly advantageous in combating drug resistance and heterogeneous tumor behavior (Kumar 2015). In contrast to single‐target drugs, multitargeted agents often achieve greater therapeutic efficacy with lower toxicity. The relatively low IC₅₀ value in breast cancer cells, coupled with a higher tolerance in MCF‐10A cells, underscores this favorable pharmacodynamic balance (Kifle et al. 2021).

**Scheme 1:**
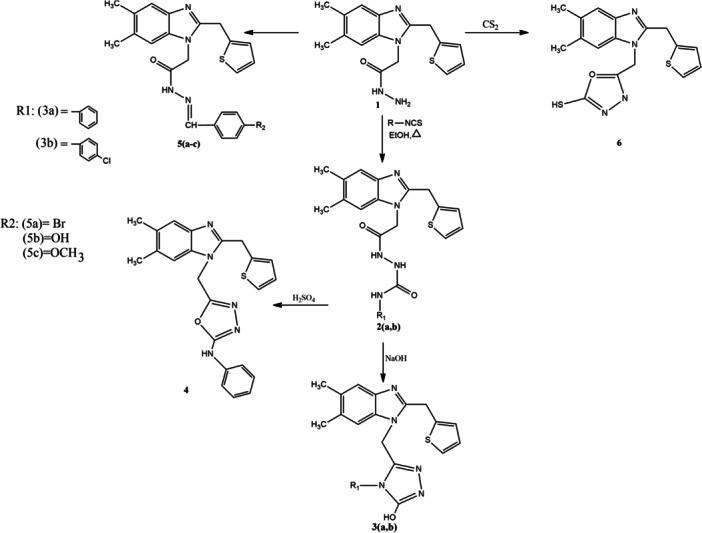
Synthetic route for the preparation of the target compounds.

Overall, the integrated experimental and computational findings establish a coherent mechanistic framework for compound 4. Its distinct benzimidazole–oxadiazole hybrid structure appears to promote both S‐phase arrest and apoptosis through concurrent inhibition of Bcl‐2/Bcl‐xL anti‐apoptotic signaling and suppression of CDK2‐Cyclin B1‐mediated cell‐cycle progression (Nazreen et al. 2022). Future studies should explore downstream biochemical markers, such as caspase activation and mitochondrial membrane depolarization, to confirm these pathways. Additionally, in vivo pharmacokinetic and toxicity evaluations will be essential to translate these promising in vitro results into potential clinical applications. Scheme 1

In conclusion, compound 4 represents a chemically and biologically optimized benzimidazole derivative with dual activity on apoptosis and cell‐cycle regulation. Its multitarget binding profile, combined with selective cytotoxicity toward cancer cells, positions it as a strong candidate for further preclinical development as a new generation anticancer scaffold (Table 3).

**Table 3:** MM‐GBSA binding free energy analysis of compound 4.

Target protein	ΔE_vdW_ (kcal/mol)	ΔE_vdW_ (kcal/mol)	ΔE_vdW_ (kcal/mol)	ΔE_vdW_ (kcal/mol)
**Bcl‐2**	−48.7	−21.3	+24.9	−45.1
**Bcl‐xL**	−44.2	−18.6	+23.4	−39.4
**CDK2**	−51.8	−24.1	+27.6	−48.3
**Cyclin E**	−42.5	−17.9	+22.8	−37.6

*Note:* [ΔG_bind_ = ΔE_vdW_ + ΔE_ele_ + ΔG_solv_].

## Funding

The authors received no specific funding for this work.

## Conflicts of Interest

The authors declare no conflicts of interest.

## Supporting information


**Table S1:** Predicted ADMET properties of compound 4 obtained using in silico tools.

Supplementary Material.

## Data Availability

The data generated for the current study are available from the corresponding author on reasonable request.
