# Supplementary material for: Synthesis, Characterization, and Biological Evaluation of Aliphatic‐Substituted Benzimidazole Derivatives: Induction of Apoptosis, Cell Cycle Arrest, and Molecular Docking in Breast Cancer Cells
Source: Drug Dev Res. 2026 Mar 29;87(2):e70267. doi: 10.1002/ddr.70267 (PMC13033344; doi:10.1002/ddr.70267)
Supplement: Supplementary file 1 — Table S1: Predicted ADMET properties of compound 4 obtained using in silico tools. [file DDR-87-e70267-s001.docx]

| **Parameter** | **Prediction** | **Interpretation** |
| --- | --- | --- |
| Molecular weight | <500 Da | Drug-like |
| cLogP | Moderate | Favorable lipophilicity |
| GI absorption | High | Potential oral bioavailability |
| Caco-2 permeability | Moderate | Adequate intestinal permeability |
| P-gp substrate | No | Reduced efflux liability |
| BBB permeability | Low | Limited CNS exposure |
| Plasma protein binding | Moderate | Acceptable free drug fraction |
| CYP3A4 inhibition | No | Low drug–drug interaction risk |
| CYP2D6 inhibition | No | Favorable metabolic profile |
| Total clearance | Moderate | Balanced elimination |
| Ames toxicity | Negative | Non-mutagenic |
| hERG inhibition | Low risk | Reduced cardiotoxicity |
| Hepatotoxicity | No | Favorable liver safety |

**Supplementary Table S1.** Predicted ADMET properties of compound 4 obtained using in silico tools.
