# Supplementary material for: Synthesis, Characterization, and Biological Evaluation of Aliphatic‐Substituted Benzimidazole Derivatives: Induction of Apoptosis, Cell Cycle Arrest, and Molecular Docking in Breast Cancer Cells
Source: Drug Dev Res. 2026 Mar 29;87(2):e70267. doi: 10.1002/ddr.70267 (PMC13033344; doi:10.1002/ddr.70267)
Supplement: Supplementary file 2 — Supplementary Material. [file DDR-87-e70267-s002.docx]

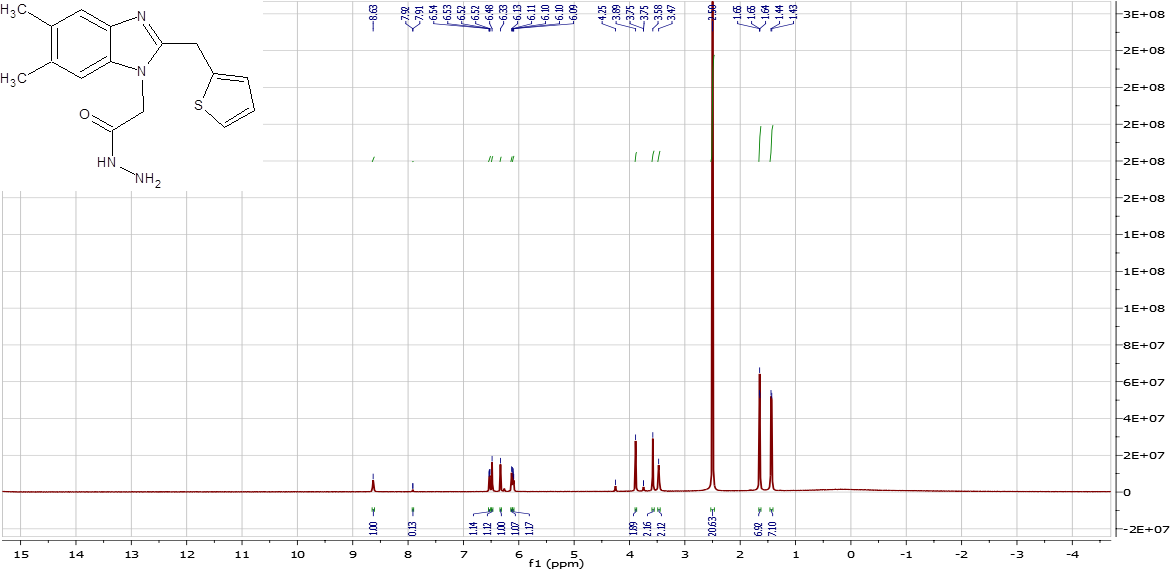


1 Compound ^1^H NMR Spectrum, 400 MHz, DMSO-d6


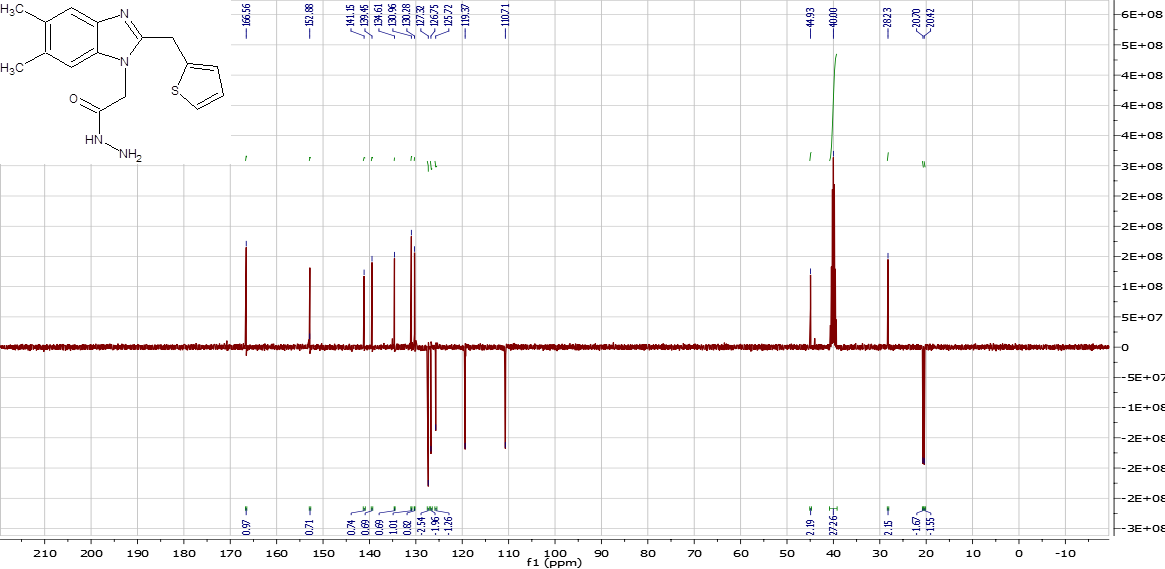


1 Compound ^13^C APT NMR Spectrum, 100 MHz, DMSO-d6

1 Compound ^1^H NMR Spectrum, 100 MHz, DMSO-d6

1 Compound ^1^H NMR Spectrum, 100 MHz, DMSO-d6

1 Compound ^1^H NMR Spectrum, 100 MHz, DMSO-d6

1 Compound ^1^H NMR Spectrum, 100 MHz, DMSO-d6

1 Compound ^1^H NMR Spectrum, 100 MHz, DMSO-d6

1 Compound ^1^H NMR Spectrum, 100 MHz, DMSO-d6

1 Compound ^1^H NMR Spectrum, 100 MHz, DMSO-d6

1 Compound ^1^H NMR Spectrum, 100 MHz, DMSO-d6

1 Compound ^1^H NMR Spectrum, 100 MHz, DMSO-d6

1 Compound ^1^H NMR Spectrum, 100 MHz, DMSO-d6

1 Compound ^1^H NMR Spectrum, 100 MHz, DMSO-d6

1 Compound ^1^H NMR Spectrum, 100 MHz, DMSO-d6

1 Compound ^1^H NMR Spectrum, 100 MHz, DMSO-d6

1 Compound ^1^H NMR Spectrum, 100 MHz, DMSO-d6

1 Compound ^1^H NMR Spectrum, 100 MHz, DMSO-d6

1 Compound ^1^H NMR Spectrum, 100 MHz, DMSO-d6

1 Compound ^1^H NMR Spectrum, 100 MHz, DMSO-d6

1 Compound ^1^H NMR Spectrum, 100 MHz, DMSO-d6

1 Compound ^1^H NMR Spectrum, 100 MHz, DMSO-d6

1 Compound ^1^H NMR Spectrum, 100 MHz, DMSO-d6

1 Compound ^1^H NMR Spectrum, 100 MHz, DMSO-d6

1 Compound ^1^H NMR Spectrum, 100 MHz, DMSO-d6

1 Compound ^1^H NMR Spectrum, 100 MHz, DMSO-d6

1 Compound ^1^H NMR Spectrum, 100 MHz, DMSO-d6

1 Compound ^1^H NMR Spectrum, 100 MHz, DMSO-d6

1 Compound ^1^H NMR Spectrum, 100 MHz, DMSO-d6

1 Compound ^1^H NMR Spectrum, 100 MHz, DMSO-d6

1 Compound ^1^H NMR Spectrum, 100 MHz, DMSO-d6

1 Compound ^1^H NMR Spectrum, 100 MHz, DMSO-d6

1 Compound ^1^H NMR Spectrum, 100 MHz, DMSO-d6

1 Compound ^1^H NMR Spectrum, 100 MHz, DMSO-d6


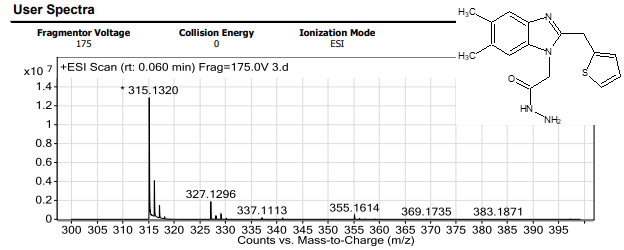


1 Compound LC/TOF-MS Spectrum

1 Compound Kütle Spectrum

1 Compound Kütle Spectrum

1 Compound Kütle Spectrum

1 Compound Kütle Spectrum

1 Compound Kütle Spectrum

1 Compound Kütle Spectrum

1 Compound Kütle Spectrum

1 Compound Kütle Spectrum

1 Compound Kütle Spectrum

1 Compound Kütle Spectrum

1 Compound Kütle Spectrum

1 Compound Kütle Spectrum

1 Compound Kütle Spectrum

1 Compound Kütle Spectrum

1 Compound Kütle Spectrum

1 Compound Kütle Spectrum

1 Compound Kütle Spectrum

1 Compound Kütle Spectrum

1 Compound Kütle Spectrum

1 Compound Kütle Spectrum

1 Compound Kütle Spectrum

1 Compound Kütle Spectrum

1 Compound Kütle Spectrum

1 Compound Kütle Spectrum

1 Compound Kütle Spectrum

1 Compound Kütle Spectrum

1 Compound Kütle Spectrum

1 Compound Kütle Spectrum

1 Compound Kütle Spectrum

1 Compound Kütle Spectrum

1 Compound Kütle Spectrum


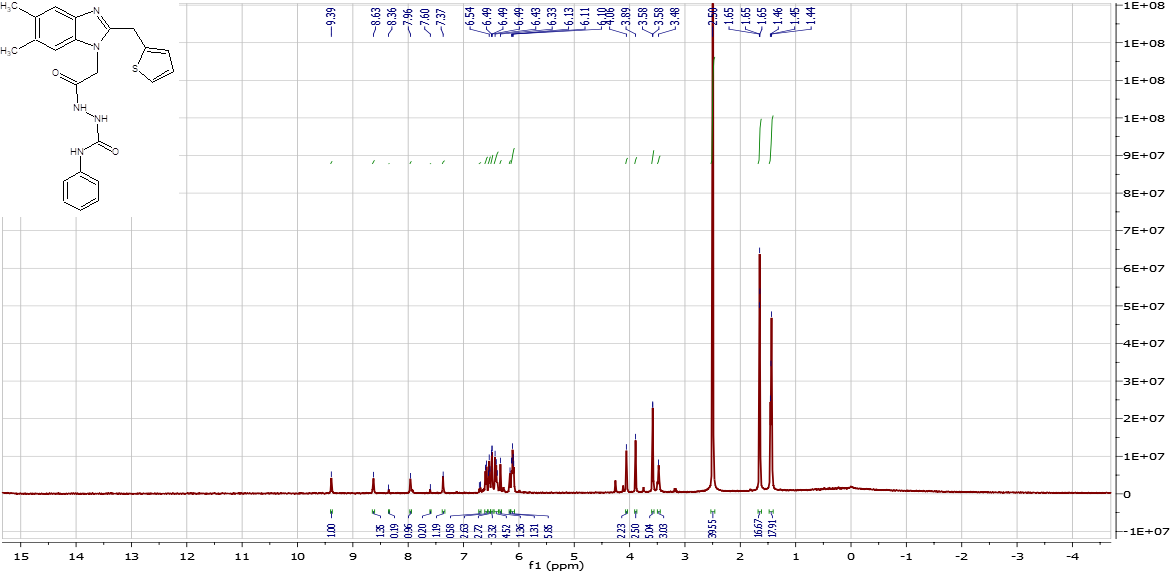


2a Compound ^1^H NMR Spectrum, 400 MHz, DMSO-d6


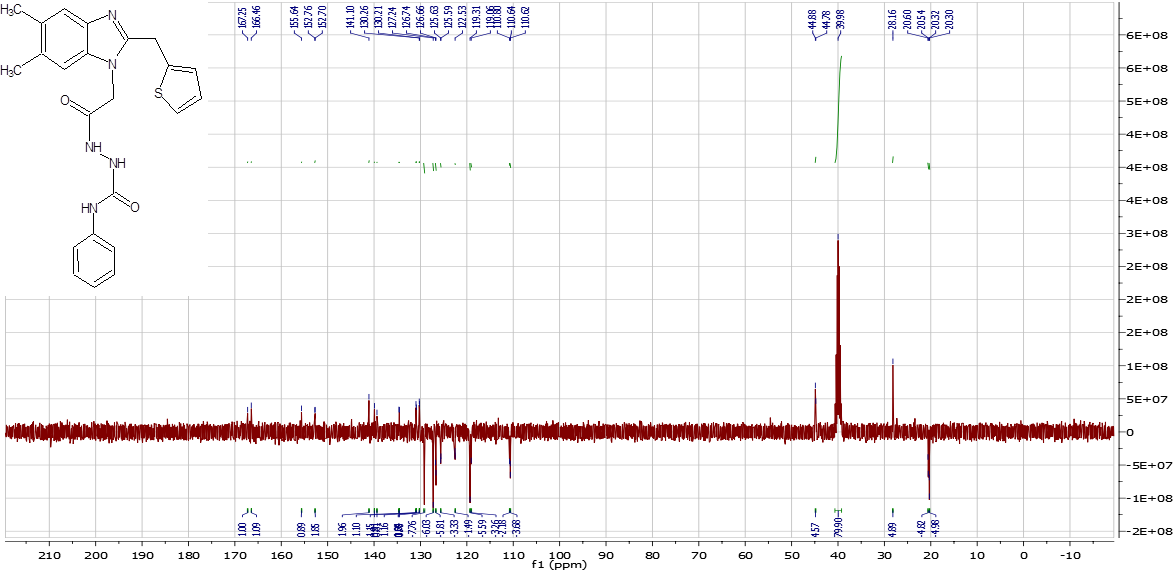


2a Compound ^13^C APT NMR Spectrum, 100 MHz, DMSO-d6

1 Compound ^1^H NMR Spectrum, 100 MHz, DMSO-d6

1 Compound ^1^H NMR Spectrum, 100 MHz, DMSO-d6

1 Compound ^1^H NMR Spectrum, 100 MHz, DMSO-d6

1 Compound ^1^H NMR Spectrum, 100 MHz, DMSO-d6

1 Compound ^1^H NMR Spectrum, 100 MHz, DMSO-d6

1 Compound ^1^H NMR Spectrum, 100 MHz, DMSO-d6

1 Compound ^1^H NMR Spectrum, 100 MHz, DMSO-d6

1 Compound ^1^H NMR Spectrum, 100 MHz, DMSO-d6

1 Compound ^1^H NMR Spectrum, 100 MHz, DMSO-d6

1 Compound ^1^H NMR Spectrum, 100 MHz, DMSO-d6

1 Compound ^1^H NMR Spectrum, 100 MHz, DMSO-d6

1 Compound ^1^H NMR Spectrum, 100 MHz, DMSO-d6

1 Compound ^1^H NMR Spectrum, 100 MHz, DMSO-d6

1 Compound ^1^H NMR Spectrum, 100 MHz, DMSO-d6

1 Compound ^1^H NMR Spectrum, 100 MHz, DMSO-d6

1 Compound ^1^H NMR Spectrum, 100 MHz, DMSO-d6

1 Compound ^1^H NMR Spectrum, 100 MHz, DMSO-d6

1 Compound ^1^H NMR Spectrum, 100 MHz, DMSO-d6

1 Compound ^1^H NMR Spectrum, 100 MHz, DMSO-d6

1 Compound ^1^H NMR Spectrum, 100 MHz, DMSO-d6

1 Compound ^1^H NMR Spectrum, 100 MHz, DMSO-d6

1 Compound ^1^H NMR Spectrum, 100 MHz, DMSO-d6

1 Compound ^1^H NMR Spectrum, 100 MHz, DMSO-d6

1 Compound ^1^H NMR Spectrum, 100 MHz, DMSO-d6

1 Compound ^1^H NMR Spectrum, 100 MHz, DMSO-d6

1 Compound ^1^H NMR Spectrum, 100 MHz, DMSO-d6

1 Compound ^1^H NMR Spectrum, 100 MHz, DMSO-d6

1 Compound ^1^H NMR Spectrum, 100 MHz, DMSO-d6

1 Compound ^1^H NMR Spectrum, 100 MHz, DMSO-d6

1 Compound ^1^H NMR Spectrum, 100 MHz, DMSO-d6

1 Compound ^1^H NMR Spectrum, 100 MHz, DMSO-d6


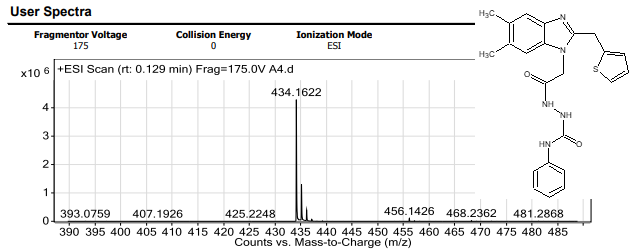


2a Compound LC/TOF-MS Spectrum

1 Compound Kütle Spectrum

1 Compound Kütle Spectrum

1 Compound Kütle Spectrum

1 Compound Kütle Spectrum

1 Compound Kütle Spectrum

1 Compound Kütle Spectrum

1 Compound Kütle Spectrum

1 Compound Kütle Spectrum

1 Compound Kütle Spectrum

1 Compound Kütle Spectrum

1 Compound Kütle Spectrum

1 Compound Kütle Spectrum

1 Compound Kütle Spectrum

1 Compound Kütle Spectrum

1 Compound Kütle Spectrum

1 Compound Kütle Spectrum

1 Compound Kütle Spectrum

1 Compound Kütle Spectrum

1 Compound Kütle Spectrum

1 Compound Kütle Spectrum

1 Compound Kütle Spectrum

1 Compound Kütle Spectrum

1 Compound Kütle Spectrum

1 Compound Kütle Spectrum

1 Compound Kütle Spectrum

1 Compound Kütle Spectrum

1 Compound Kütle Spectrum

1 Compound Kütle Spectrum

1 Compound Kütle Spectrum

1 Compound Kütle Spectrum

1 Compound Kütle Spectrum


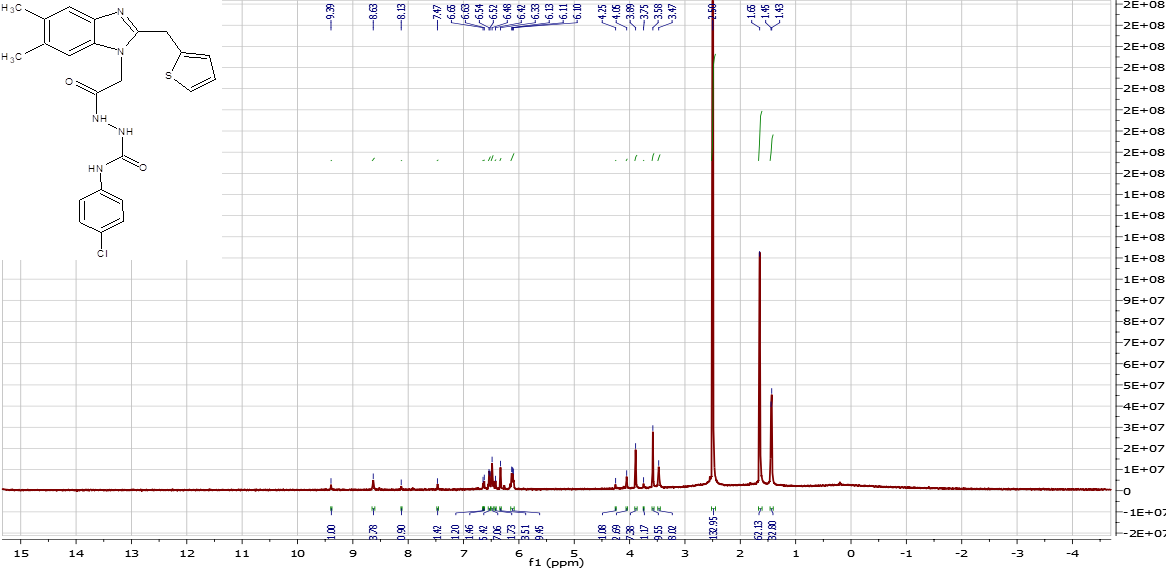


2b Compound ^1^H NMR Spectrum, 400 MHz, DMSO-d6


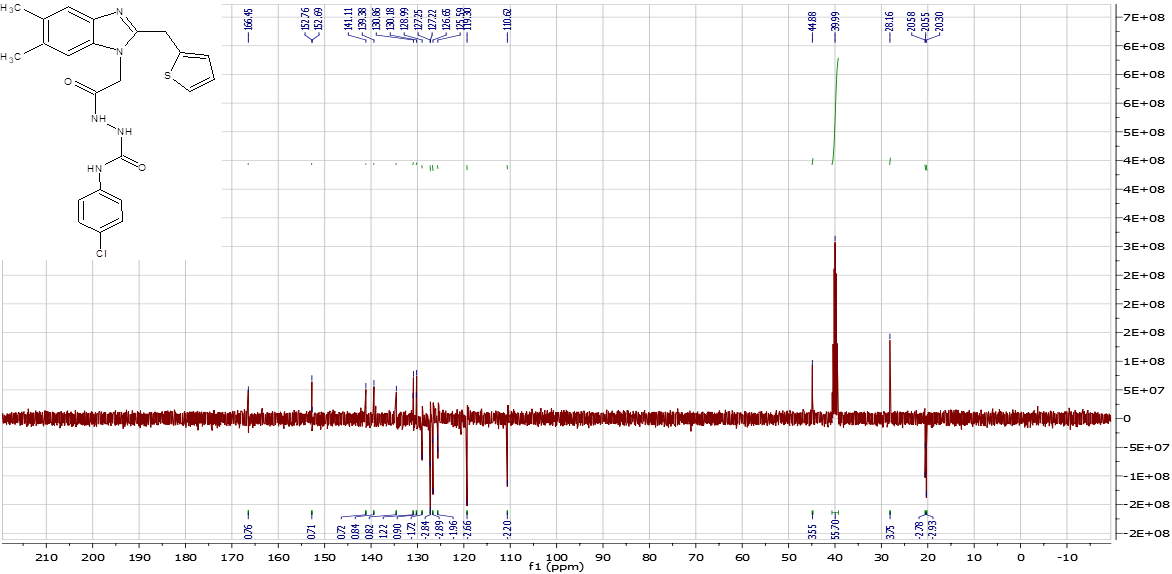


2b Compound ^13^C APT NMR Spectrum, 100 MHz, DMSO-d6

1 Compound ^1^H NMR Spectrum, 100 MHz, DMSO-d6

1 Compound ^1^H NMR Spectrum, 100 MHz, DMSO-d6

1 Compound ^1^H NMR Spectrum, 100 MHz, DMSO-d6

1 Compound ^1^H NMR Spectrum, 100 MHz, DMSO-d6

1 Compound ^1^H NMR Spectrum, 100 MHz, DMSO-d6

1 Compound ^1^H NMR Spectrum, 100 MHz, DMSO-d6

1 Compound ^1^H NMR Spectrum, 100 MHz, DMSO-d6

1 Compound ^1^H NMR Spectrum, 100 MHz, DMSO-d6

1 Compound ^1^H NMR Spectrum, 100 MHz, DMSO-d6

1 Compound ^1^H NMR Spectrum, 100 MHz, DMSO-d6

1 Compound ^1^H NMR Spectrum, 100 MHz, DMSO-d6

1 Compound ^1^H NMR Spectrum, 100 MHz, DMSO-d6

1 Compound ^1^H NMR Spectrum, 100 MHz, DMSO-d6

1 Compound ^1^H NMR Spectrum, 100 MHz, DMSO-d6

1 Compound ^1^H NMR Spectrum, 100 MHz, DMSO-d6

1 Compound ^1^H NMR Spectrum, 100 MHz, DMSO-d6

1 Compound ^1^H NMR Spectrum, 100 MHz, DMSO-d6

1 Compound ^1^H NMR Spectrum, 100 MHz, DMSO-d6

1 Compound ^1^H NMR Spectrum, 100 MHz, DMSO-d6

1 Compound ^1^H NMR Spectrum, 100 MHz, DMSO-d6

1 Compound ^1^H NMR Spectrum, 100 MHz, DMSO-d6

1 Compound ^1^H NMR Spectrum, 100 MHz, DMSO-d6

1 Compound ^1^H NMR Spectrum, 100 MHz, DMSO-d6

1 Compound ^1^H NMR Spectrum, 100 MHz, DMSO-d6

1 Compound ^1^H NMR Spectrum, 100 MHz, DMSO-d6

1 Compound ^1^H NMR Spectrum, 100 MHz, DMSO-d6

1 Compound ^1^H NMR Spectrum, 100 MHz, DMSO-d6

1 Compound ^1^H NMR Spectrum, 100 MHz, DMSO-d6

1 Compound ^1^H NMR Spectrum, 100 MHz, DMSO-d6

1 Compound ^1^H NMR Spectrum, 100 MHz, DMSO-d6

1 Compound ^1^H NMR Spectrum, 100 MHz, DMSO-d6


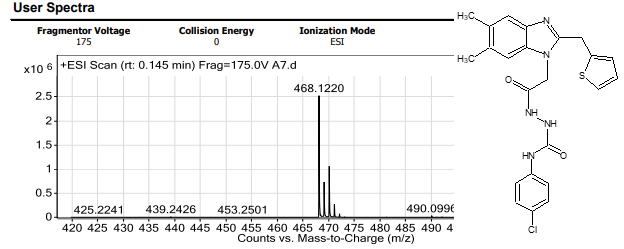


2b Compound LC/TOF-MS Spectrum

1 Compound Spectrum

1 Compound Spectrum

1 Compound Spectrum

1 Compound Spectrum

1 Compound Spectrum

1 Compound Spectrum

1 Compound Spectrum

1 Compound Spectrum

1 Compound Spectrum

1 Compound Spectrum

1 Compound Spectrum

1 Compound Spectrum

1 Compound Spectrum

1 Compound Spectrum

1 Compound Spectrum

1 Compound Spectrum

1 Compound Spectrum

1 Compound Spectrum

1 Compound Spectrum

1 Compound Spectrum

1 Compound Spectrum

1 Compound Spectrum

1 Compound Spectrum

1 Compound Spectrum

1 Compound Spectrum

1 Compound Spectrum

1 Compound Spectrum

1 Compound Spectrum

1 Compound Spectrum

1 Compound Spectrum

1 Compound Spectrum


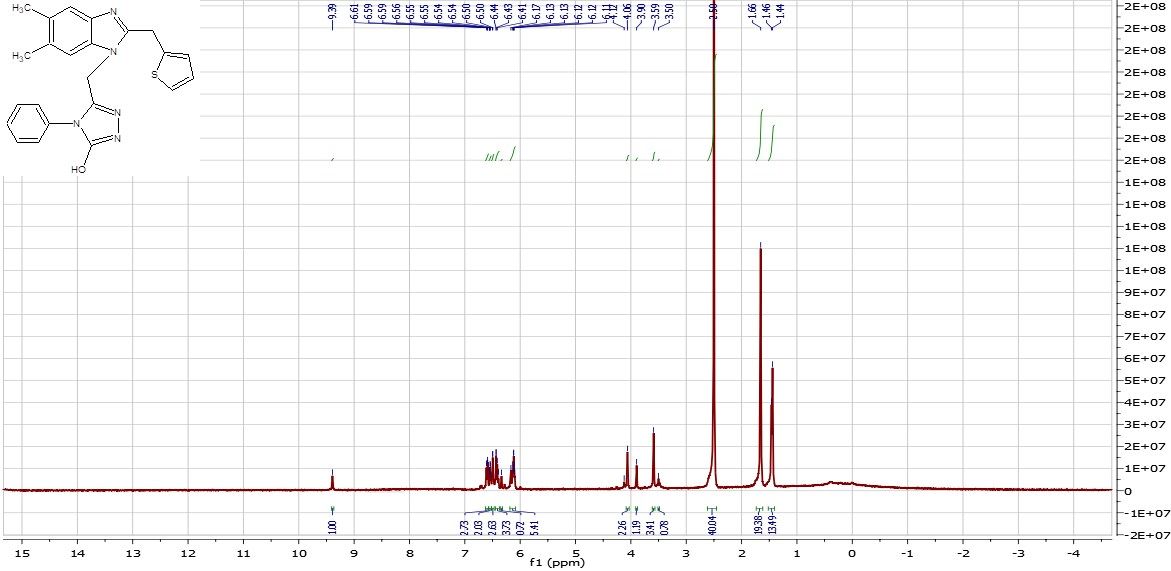


3a Compound ^1^H NMR Spectrum, 400 MHz, DMSO-d6


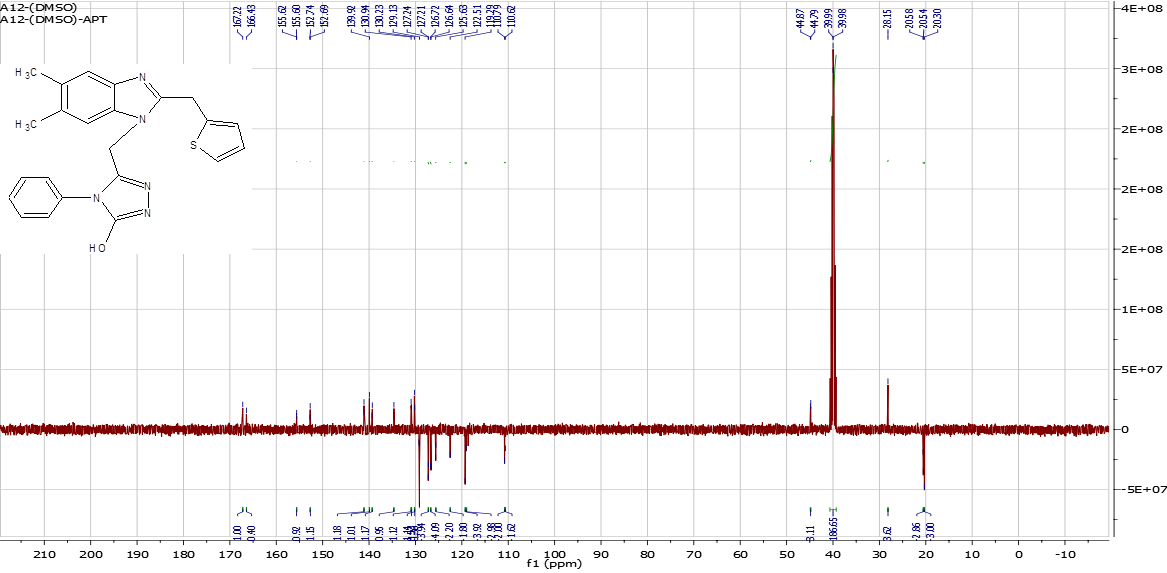


3a Compound ^13^C APT NMR Spectrum, 100 MHz, DMSO-d6

1 Compound ^1^H NMR Spectrum, 100 MHz, DMSO-d6

1 Compound ^1^H NMR Spectrum, 100 MHz, DMSO-d6

1 Compound ^1^H NMR Spectrum, 100 MHz, DMSO-d6

1 Compound ^1^H NMR Spectrum, 100 MHz, DMSO-d6

1 Compound ^1^H NMR Spectrum, 100 MHz, DMSO-d6

1 Compound ^1^H NMR Spectrum, 100 MHz, DMSO-d6

1 Compound ^1^H NMR Spectrum, 100 MHz, DMSO-d6

1 Compound ^1^H NMR Spectrum, 100 MHz, DMSO-d6

1 Compound ^1^H NMR Spectrum, 100 MHz, DMSO-d6

1 Compound ^1^H NMR Spectrum, 100 MHz, DMSO-d6

1 Compound ^1^H NMR Spectrum, 100 MHz, DMSO-d6

1 Compound ^1^H NMR Spectrum, 100 MHz, DMSO-d6

1 Compound ^1^H NMR Spectrum, 100 MHz, DMSO-d6

1 Compound ^1^H NMR Spectrum, 100 MHz, DMSO-d6

1 Compound ^1^H NMR Spectrum, 100 MHz, DMSO-d6

1 Compound ^1^H NMR Spectrum, 100 MHz, DMSO-d6

1 Compound ^1^H NMR Spectrum, 100 MHz, DMSO-d6

1 Compound ^1^H NMR Spectrum, 100 MHz, DMSO-d6

1 Compound ^1^H NMR Spectrum, 100 MHz, DMSO-d6

1 Compound ^1^H NMR Spectrum, 100 MHz, DMSO-d6

1 Compound ^1^H NMR Spectrum, 100 MHz, DMSO-d6

1 Compound ^1^H NMR Spectrum, 100 MHz, DMSO-d6

1 Compound ^1^H NMR Spectrum, 100 MHz, DMSO-d6

1 Compound ^1^H NMR Spectrum, 100 MHz, DMSO-d6

1 Compound ^1^H NMR Spectrum, 100 MHz, DMSO-d6

1 Compound ^1^H NMR Spectrum, 100 MHz, DMSO-d6

1 Compound ^1^H NMR Spectrum, 100 MHz, DMSO-d6

1 Compound ^1^H NMR Spectrum, 100 MHz, DMSO-d6

1 Compound ^1^H NMR Spectrum, 100 MHz, DMSO-d6

1 Compound ^1^H NMR Spectrum, 100 MHz, DMSO-d6

1 Compound ^1^H NMR Spectrum, 100 MHz, DMSO-d6


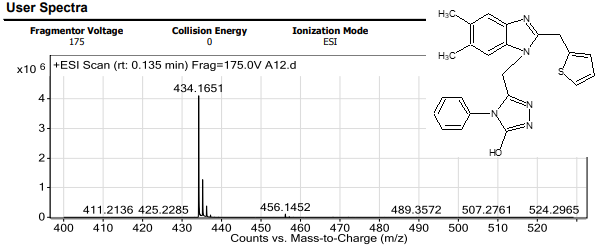


3a Compound LC/TOF-MS Spectrum

1 Compound Spectrum

1 Compound Spectrum

1 Compound Spectrum

1 Compound Spectrum

1 Compound Spectrum

1 Compound Spectrum

1 Compound Spectrum

1 Compound Spectrum

1 Compound Spectrum

1 Compound Spectrum

1 Compound Spectrum

1 Compound Spectrum

1 Compound Spectrum

1 Compound Spectrum

1 Compound Spectrum

1 Compound Spectrum

1 Compound Spectrum

1 Compound Spectrum

1 Compound Spectrum

1 Compound Spectrum

1 Compound Spectrum

1 Compound Spectrum

1 Compound Spectrum

1 Compound Spectrum

1 Compound Spectrum

1 Compound Spectrum

1 Compound Spectrum

1 Compound Spectrum

1 Compound Spectrum

1 Compound Spectrum

1 Compound Spectrum


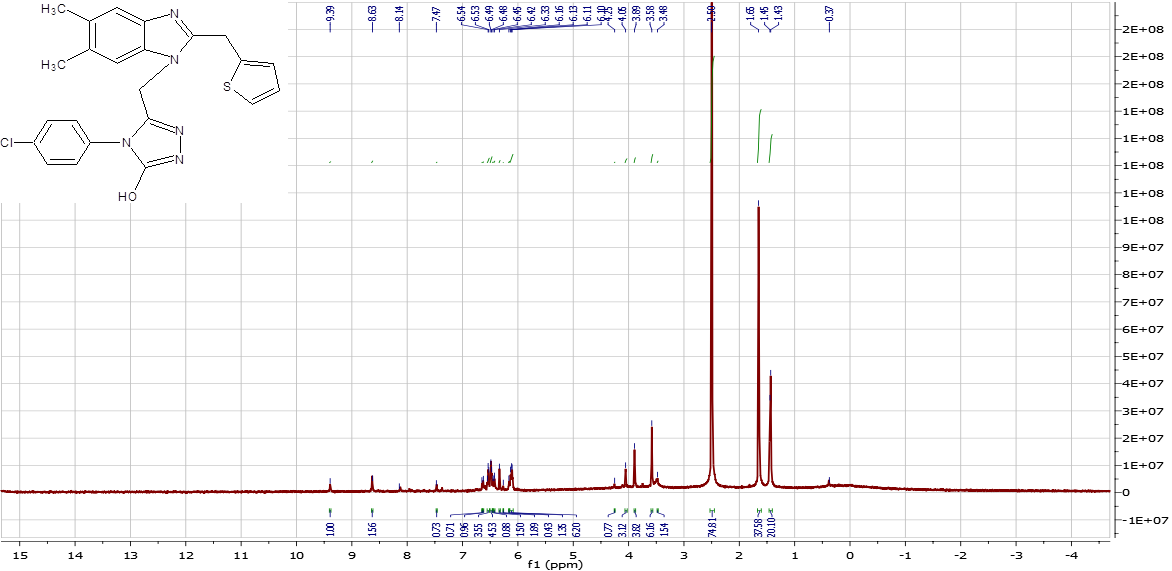


3b Compound ^1^H NMR Spectrum, 400 MHz, DMSO-d6


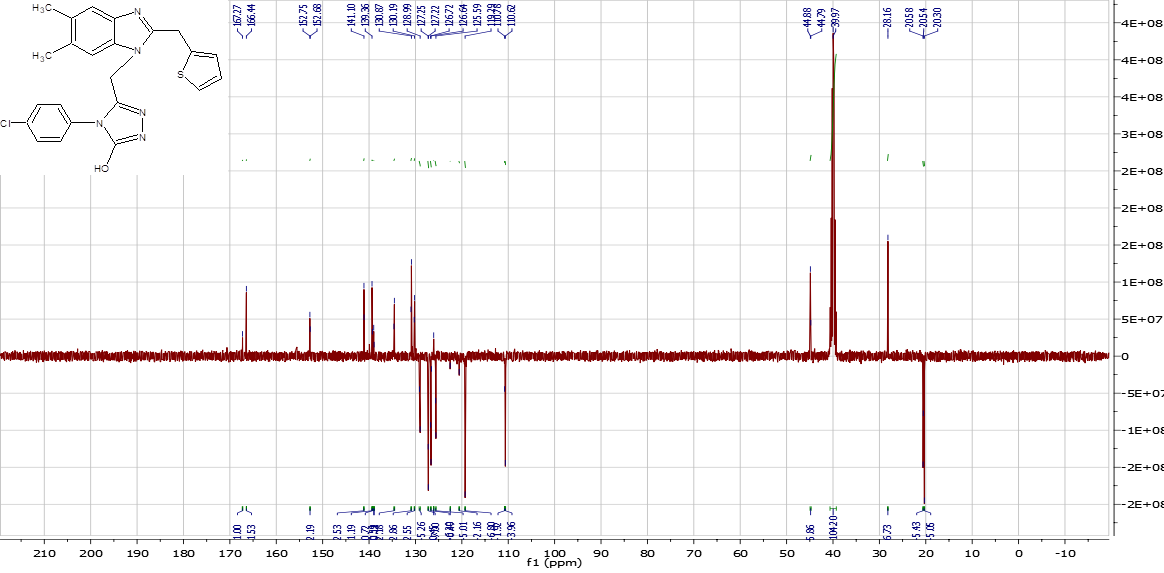


3b Compound ^13^C APT NMR Spectrum, 100 MHz, DMSO-d6

1 Compound ^1^H NMR Spectrum, 100 MHz, DMSO-d6

1 Compound ^1^H NMR Spectrum, 100 MHz, DMSO-d6

1 Compound ^1^H NMR Spectrum, 100 MHz, DMSO-d6

1 Compound ^1^H NMR Spectrum, 100 MHz, DMSO-d6

1 Compound ^1^H NMR Spectrum, 100 MHz, DMSO-d6

1 Compound ^1^H NMR Spectrum, 100 MHz, DMSO-d6

1 Compound ^1^H NMR Spectrum, 100 MHz, DMSO-d6

1 Compound ^1^H NMR Spectrum, 100 MHz, DMSO-d6

1 Compound ^1^H NMR Spectrum, 100 MHz, DMSO-d6

1 Compound ^1^H NMR Spectrum, 100 MHz, DMSO-d6

1 Compound ^1^H NMR Spectrum, 100 MHz, DMSO-d6

1 Compound ^1^H NMR Spectrum, 100 MHz, DMSO-d6

1 Compound ^1^H NMR Spectrum, 100 MHz, DMSO-d6

1 Compound ^1^H NMR Spectrum, 100 MHz, DMSO-d6

1 Compound ^1^H NMR Spectrum, 100 MHz, DMSO-d6

1 Compound ^1^H NMR Spectrum, 100 MHz, DMSO-d6

1 Compound ^1^H NMR Spectrum, 100 MHz, DMSO-d6

1 Compound ^1^H NMR Spectrum, 100 MHz, DMSO-d6

1 Compound ^1^H NMR Spectrum, 100 MHz, DMSO-d6

1 Compound ^1^H NMR Spectrum, 100 MHz, DMSO-d6

1 Compound ^1^H NMR Spectrum, 100 MHz, DMSO-d6

1 Compound ^1^H NMR Spectrum, 100 MHz, DMSO-d6

1 Compound ^1^H NMR Spectrum, 100 MHz, DMSO-d6

1 Compound ^1^H NMR Spectrum, 100 MHz, DMSO-d6

1 Compound ^1^H NMR Spectrum, 100 MHz, DMSO-d6

1 Compound ^1^H NMR Spectrum, 100 MHz, DMSO-d6

1 Compound ^1^H NMR Spectrum, 100 MHz, DMSO-d6

1 Compound ^1^H NMR Spectrum, 100 MHz, DMSO-d6

1 Compound ^1^H NMR Spectrum, 100 MHz, DMSO-d6

1 Compound ^1^H NMR Spectrum, 100 MHz, DMSO-d6

1 Compound ^1^H NMR Spectrum, 100 MHz, DMSO-d6


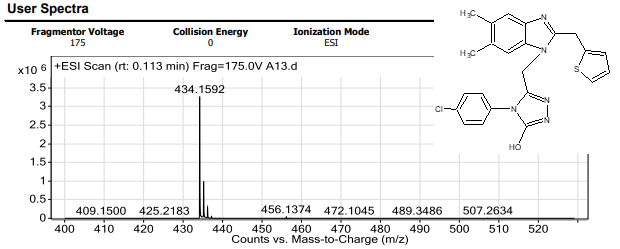


3b Compound LC/TOF-MS Spectrum

1 Compound Kütle Spectrum

1 Compound Kütle Spectrum

1 Compound Kütle Spectrum

1 Compound Kütle Spectrum

1 Compound Kütle Spectrum

1 Compound Kütle Spectrum

1 Compound Kütle Spectrum

1 Compound Kütle Spectrum

1 Compound Kütle Spectrum

1 Compound Kütle Spectrum

1 Compound Kütle Spectrum

1 Compound Kütle Spectrum

1 Compound Kütle Spectrum

1 Compound Kütle Spectrum

1 Compound Kütle Spectrum

1 Compound Kütle Spectrum

1 Compound Kütle Spectrum

1 Compound Kütle Spectrum

1 Compound Kütle Spectrum

1 Compound Kütle Spectrum

1 Compound Kütle Spectrum

1 Compound Kütle Spectrum

1 Compound Kütle Spectrum

1 Compound Kütle Spectrum

1 Compound Kütle Spectrum

1 Compound Kütle Spectrum

1 Compound Kütle Spectrum

1 Compound Kütle Spectrum

1 Compound Kütle Spectrum

1 Compound Kütle Spectrum

1 Compound Kütle Spectrum


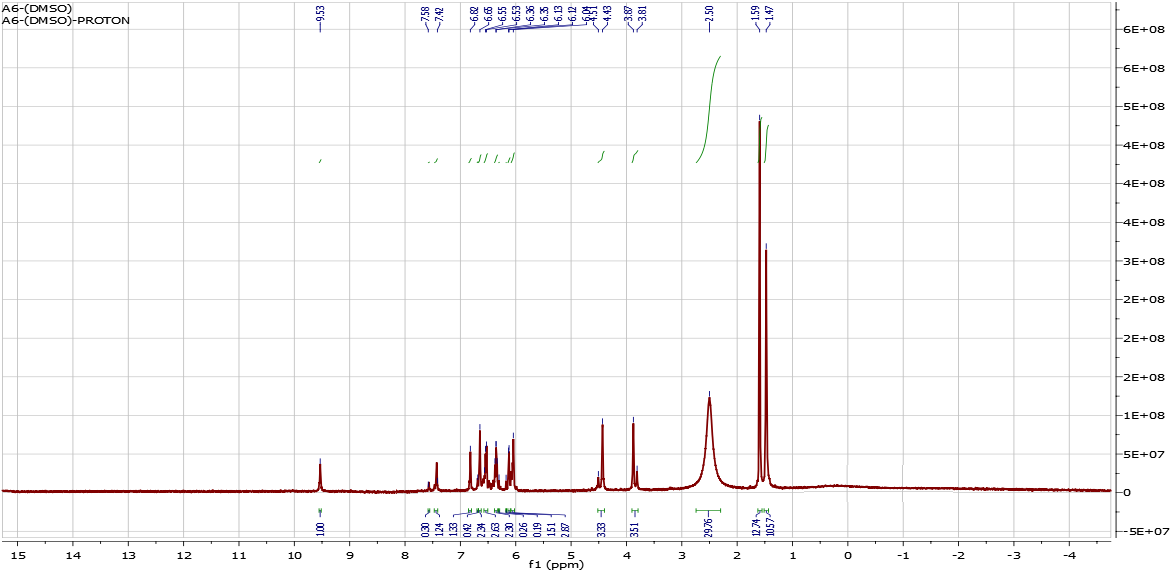


4 Compound ^1^H NMR Spectrum, 400 MHz, DMSO-d6


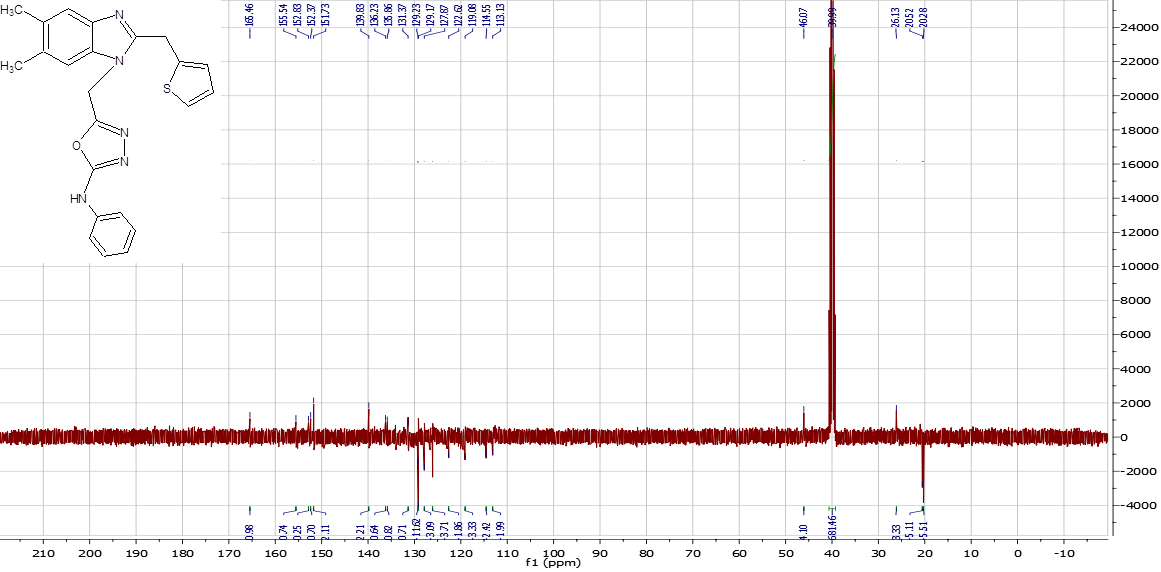


4 Compound ^13^C APT NMR Spectrum, 100 MHz, DMSO-d6

1 Compound ^1^H NMR Spectrum, 100 MHz, DMSO-d6

1 Compound ^1^H NMR Spectrum, 100 MHz, DMSO-d6

1 Compound ^1^H NMR Spectrum, 100 MHz, DMSO-d6

1 Compound ^1^H NMR Spectrum, 100 MHz, DMSO-d6

1 Compound ^1^H NMR Spectrum, 100 MHz, DMSO-d6

1 Compound ^1^H NMR Spectrum, 100 MHz, DMSO-d6

1 Compound ^1^H NMR Spectrum, 100 MHz, DMSO-d6

1 Compound ^1^H NMR Spectrum, 100 MHz, DMSO-d6

1 Compound ^1^H NMR Spectrum, 100 MHz, DMSO-d6

1 Compound ^1^H NMR Spectrum, 100 MHz, DMSO-d6

1 Compound ^1^H NMR Spectrum, 100 MHz, DMSO-d6

1 Compound ^1^H NMR Spectrum, 100 MHz, DMSO-d6

1 Compound ^1^H NMR Spectrum, 100 MHz, DMSO-d6

1 Compound ^1^H NMR Spectrum, 100 MHz, DMSO-d6

1 Compound ^1^H NMR Spectrum, 100 MHz, DMSO-d6

1 Compound ^1^H NMR Spectrum, 100 MHz, DMSO-d6

1 Compound ^1^H NMR Spectrum, 100 MHz, DMSO-d6

1 Compound ^1^H NMR Spectrum, 100 MHz, DMSO-d6

1 Compound ^1^H NMR Spectrum, 100 MHz, DMSO-d6

1 Compound ^1^H NMR Spectrum, 100 MHz, DMSO-d6

1 Compound ^1^H NMR Spectrum, 100 MHz, DMSO-d6

1 Compound ^1^H NMR Spectrum, 100 MHz, DMSO-d6

1 Compound ^1^H NMR Spectrum, 100 MHz, DMSO-d6

1 Compound ^1^H NMR Spectrum, 100 MHz, DMSO-d6

1 Compound ^1^H NMR Spectrum, 100 MHz, DMSO-d6

1 Compound ^1^H NMR Spectrum, 100 MHz, DMSO-d6

1 Compound ^1^H NMR Spectrum, 100 MHz, DMSO-d6

1 Compound ^1^H NMR Spectrum, 100 MHz, DMSO-d6

1 Compound ^1^H NMR Spectrum, 100 MHz, DMSO-d6

1 Compound ^1^H NMR Spectrum, 100 MHz, DMSO-d6

1 Compound ^1^H NMR Spectrum, 100 MHz, DMSO-d6


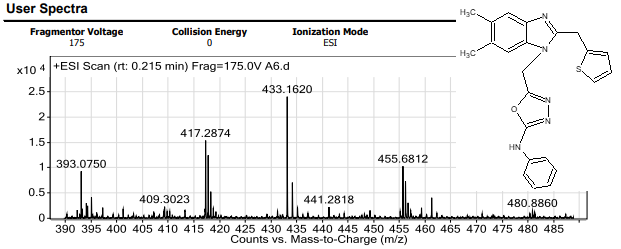


4 Compound LC/TOF-MS Spectrum

1 Compound Kütle Spectrum

1 Compound Kütle Spectrum

1 Compound Kütle Spectrum

1 Compound Kütle Spectrum

1 Compound Kütle Spectrum

1 Compound Kütle Spectrum

1 Compound Kütle Spectrum

1 Compound Kütle Spectrum

1 Compound Kütle Spectrum

1 Compound Kütle Spectrum

1 Compound Kütle Spectrum

1 Compound Kütle Spectrum

1 Compound Kütle Spectrum

1 Compound Kütle Spectrum

1 Compound Kütle Spectrum

1 Compound Kütle Spectrum

1 Compound Kütle Spectrum

1 Compound Kütle Spectrum

1 Compound Kütle Spectrum

1 Compound Kütle Spectrum

1 Compound Kütle Spectrum

1 Compound Kütle Spectrum

1 Compound Kütle Spectrum

1 Compound Kütle Spectrum

1 Compound Kütle Spectrum

1 Compound Kütle Spectrum

1 Compound Kütle Spectrum

1 Compound Kütle Spectrum

1 Compound Kütle Spectrum

1 Compound Kütle Spectrum

1 Compound Kütle Spectrum


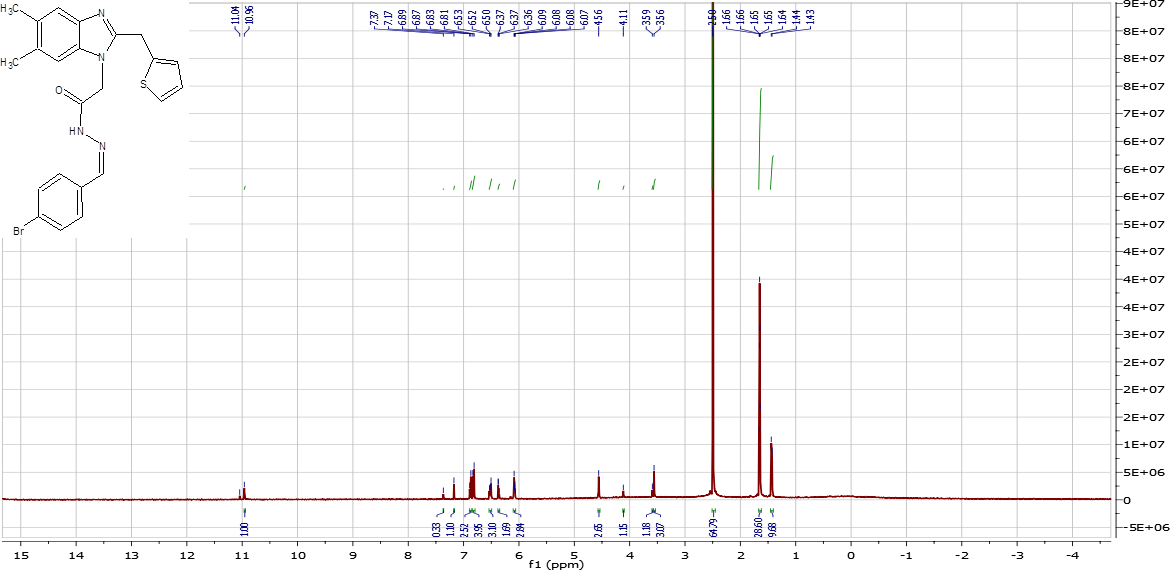


5a Compound ^1^H NMR Spectrum, 400 MHz, DMSO-d6


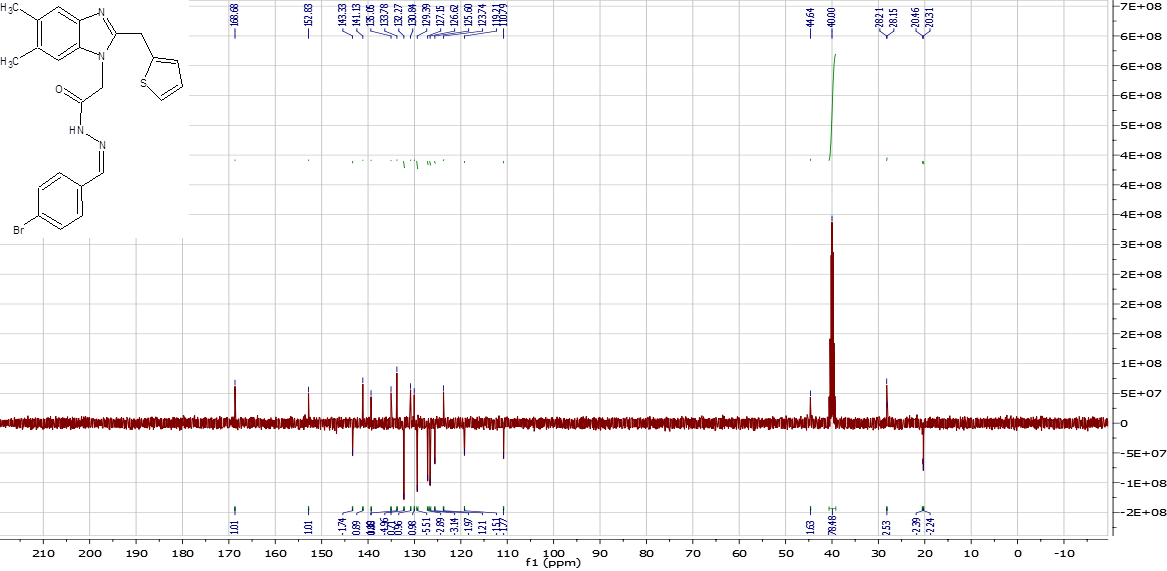


5a Compound ^13^C APT NMR Spectrum, 100 MHz, DMSO-d6

1 Compound ^1^H NMR Spectrum, 100 MHz, DMSO-d6

1 Compound ^1^H NMR Spectrum, 100 MHz, DMSO-d6

1 Compound ^1^H NMR Spectrum, 100 MHz, DMSO-d6

1 Compound ^1^H NMR Spectrum, 100 MHz, DMSO-d6

1 Compound ^1^H NMR Spectrum, 100 MHz, DMSO-d6

1 Compound ^1^H NMR Spectrum, 100 MHz, DMSO-d6

1 Compound ^1^H NMR Spectrum, 100 MHz, DMSO-d6

1 Compound ^1^H NMR Spectrum, 100 MHz, DMSO-d6

1 Compound ^1^H NMR Spectrum, 100 MHz, DMSO-d6

1 Compound ^1^H NMR Spectrum, 100 MHz, DMSO-d6

1 Compound ^1^H NMR Spectrum, 100 MHz, DMSO-d6

1 Compound ^1^H NMR Spectrum, 100 MHz, DMSO-d6

1 Compound ^1^H NMR Spectrum, 100 MHz, DMSO-d6

1 Compound ^1^H NMR Spectrum, 100 MHz, DMSO-d6

1 Compound ^1^H NMR Spectrum, 100 MHz, DMSO-d6

1 Compound ^1^H NMR Spectrum, 100 MHz, DMSO-d6

1 Compound ^1^H NMR Spectrum, 100 MHz, DMSO-d6

1 Compound ^1^H NMR Spectrum, 100 MHz, DMSO-d6

1 Compound ^1^H NMR Spectrum, 100 MHz, DMSO-d6

1 Compound ^1^H NMR Spectrum, 100 MHz, DMSO-d6

1 Compound ^1^H NMR Spectrum, 100 MHz, DMSO-d6

1 Compound ^1^H NMR Spectrum, 100 MHz, DMSO-d6

1 Compound ^1^H NMR Spectrum, 100 MHz, DMSO-d6

1 Compound ^1^H NMR Spectrum, 100 MHz, DMSO-d6

1 Compound ^1^H NMR Spectrum, 100 MHz, DMSO-d6

1 Compound ^1^H NMR Spectrum, 100 MHz, DMSO-d6

1 Compound ^1^H NMR Spectrum, 100 MHz, DMSO-d6

1 Compound ^1^H NMR Spectrum, 100 MHz, DMSO-d6

1 Compound ^1^H NMR Spectrum, 100 MHz, DMSO-d6

1 Compound ^1^H NMR Spectrum, 100 MHz, DMSO-d6

1 Compound ^1^H NMR Spectrum, 100 MHz, DMSO-d6


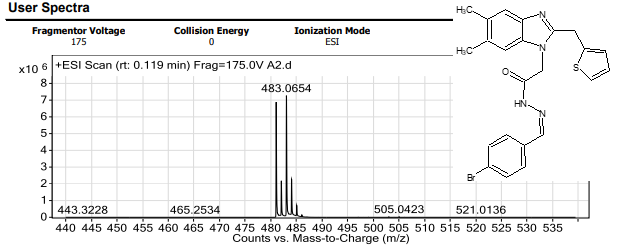


5a Compound LC/TOF-MS Spectrum

1 Compound Kütle Spectrum

1 Compound Kütle Spectrum

1 Compound Kütle Spectrum

1 Compound Kütle Spectrum

1 Compound Kütle Spectrum

1 Compound Kütle Spectrum

1 Compound Kütle Spectrum

1 Compound Kütle Spectrum

1 Compound Kütle Spectrum

1 Compound Kütle Spectrum

1 Compound Kütle Spectrum

1 Compound Kütle Spectrum

1 Compound Kütle Spectrum

1 Compound Kütle Spectrum

1 Compound Kütle Spectrum

1 Compound Kütle Spectrum

1 Compound Kütle Spectrum

1 Compound Kütle Spectrum

1 Compound Kütle Spectrum

1 Compound Kütle Spectrum

1 Compound Kütle Spectrum

1 Compound Kütle Spectrum

1 Compound Kütle Spectrum

1 Compound Kütle Spectrum

1 Compound Kütle Spectrum

1 Compound Kütle Spectrum

1 Compound Kütle Spectrum

1 Compound Kütle Spectrum

1 Compound Kütle Spectrum

1 Compound Kütle Spectrum

1 Compound Kütle Spectrum


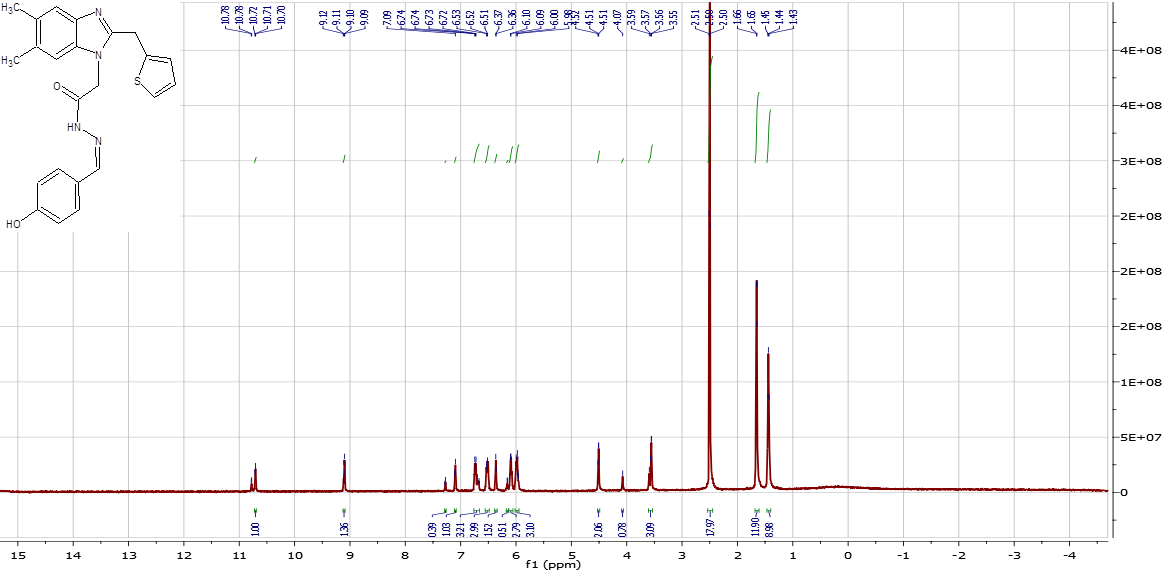


5b Compound ^1^H NMR Spectrum, 400 MHz, DMSO-d6


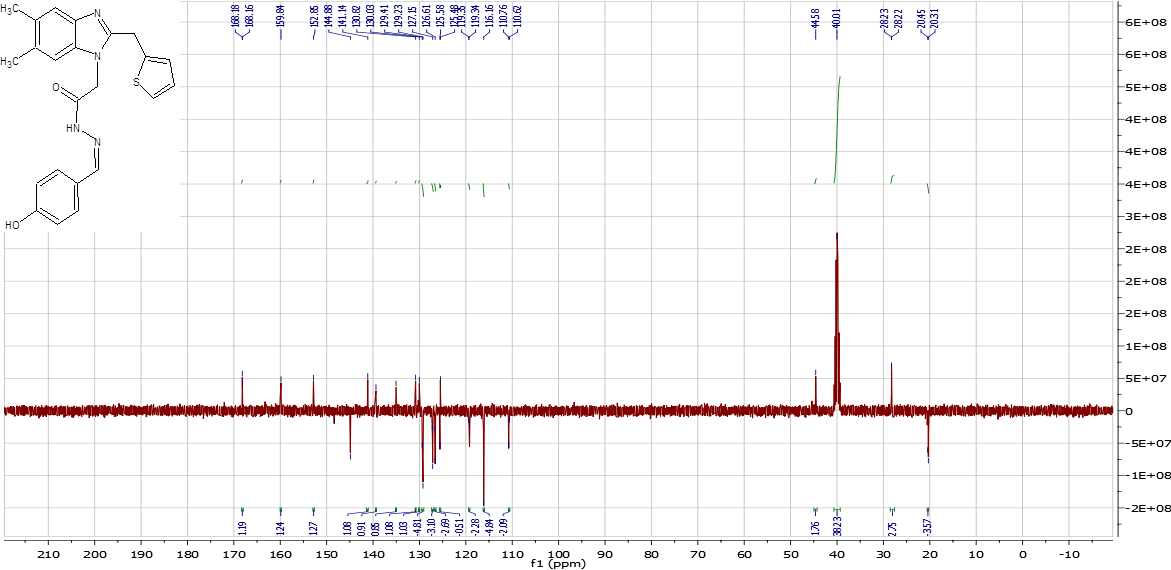


5b Compound ^13^C APT NMR Spectrum, 100 MHz, DMSO-d6

1 Compound ^1^H NMR Spectrum, 100 MHz, DMSO-d6

1 Compound ^1^H NMR Spectrum, 100 MHz, DMSO-d6

1 Compound ^1^H NMR Spectrum, 100 MHz, DMSO-d6

1 Compound ^1^H NMR Spectrum, 100 MHz, DMSO-d6

1 Compound ^1^H NMR Spectrum, 100 MHz, DMSO-d6

1 Compound ^1^H NMR Spectrum, 100 MHz, DMSO-d6

1 Compound ^1^H NMR Spectrum, 100 MHz, DMSO-d6

1 Compound ^1^H NMR Spectrum, 100 MHz, DMSO-d6

1 Compound ^1^H NMR Spectrum, 100 MHz, DMSO-d6

1 Compound ^1^H NMR Spectrum, 100 MHz, DMSO-d6

1 Compound ^1^H NMR Spectrum, 100 MHz, DMSO-d6

1 Compound ^1^H NMR Spectrum, 100 MHz, DMSO-d6

1 Compound ^1^H NMR Spectrum, 100 MHz, DMSO-d6

1 Compound ^1^H NMR Spectrum, 100 MHz, DMSO-d6

1 Compound ^1^H NMR Spectrum, 100 MHz, DMSO-d6

1 Compound ^1^H NMR Spectrum, 100 MHz, DMSO-d6

1 Compound ^1^H NMR Spectrum, 100 MHz, DMSO-d6

1 Compound ^1^H NMR Spectrum, 100 MHz, DMSO-d6

1 Compound ^1^H NMR Spectrum, 100 MHz, DMSO-d6

1 Compound ^1^H NMR Spectrum, 100 MHz, DMSO-d6

1 Compound ^1^H NMR Spectrum, 100 MHz, DMSO-d6

1 Compound ^1^H NMR Spectrum, 100 MHz, DMSO-d6

1 Compound ^1^H NMR Spectrum, 100 MHz, DMSO-d6

1 Compound ^1^H NMR Spectrum, 100 MHz, DMSO-d6

1 Compound ^1^H NMR Spectrum, 100 MHz, DMSO-d6

1 Compound ^1^H NMR Spectrum, 100 MHz, DMSO-d6

1 Compound ^1^H NMR Spectrum, 100 MHz, DMSO-d6

1 Compound ^1^H NMR Spectrum, 100 MHz, DMSO-d6

1 Compound ^1^H NMR Spectrum, 100 MHz, DMSO-d6

1 Compound ^1^H NMR Spectrum, 100 MHz, DMSO-d6

1 Compound ^1^H NMR Spectrum, 100 MHz, DMSO-d6


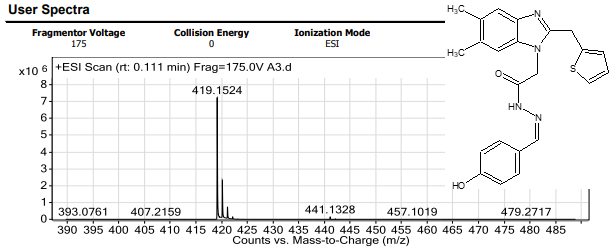


5b Compound LC/TOF-MS Spectrum

1 Compound Kütle Spectrum

1 Compound Kütle Spectrum

1 Compound Kütle Spectrum

1 Compound Kütle Spectrum

1 Compound Kütle Spectrum

1 Compound Kütle Spectrum

1 Compound Kütle Spectrum

1 Compound Kütle Spectrum

1 Compound Kütle Spectrum

1 Compound Kütle Spectrum

1 Compound Kütle Spectrum

1 Compound Kütle Spectrum

1 Compound Kütle Spectrum

1 Compound Kütle Spectrum

1 Compound Kütle Spectrum

1 Compound Kütle Spectrum

1 Compound Kütle Spectrum

1 Compound Kütle Spectrum

1 Compound Kütle Spectrum

1 Compound Kütle Spectrum

1 Compound Kütle Spectrum

1 Compound Kütle Spectrum

1 Compound Kütle Spectrum

1 Compound Kütle Spectrum

1 Compound Kütle Spectrum

1 Compound Kütle Spectrum

1 Compound Kütle Spectrum

1 Compound Kütle Spectrum

1 Compound Kütle Spectrum

1 Compound Kütle Spectrum

1 Compound Kütle Spectrum


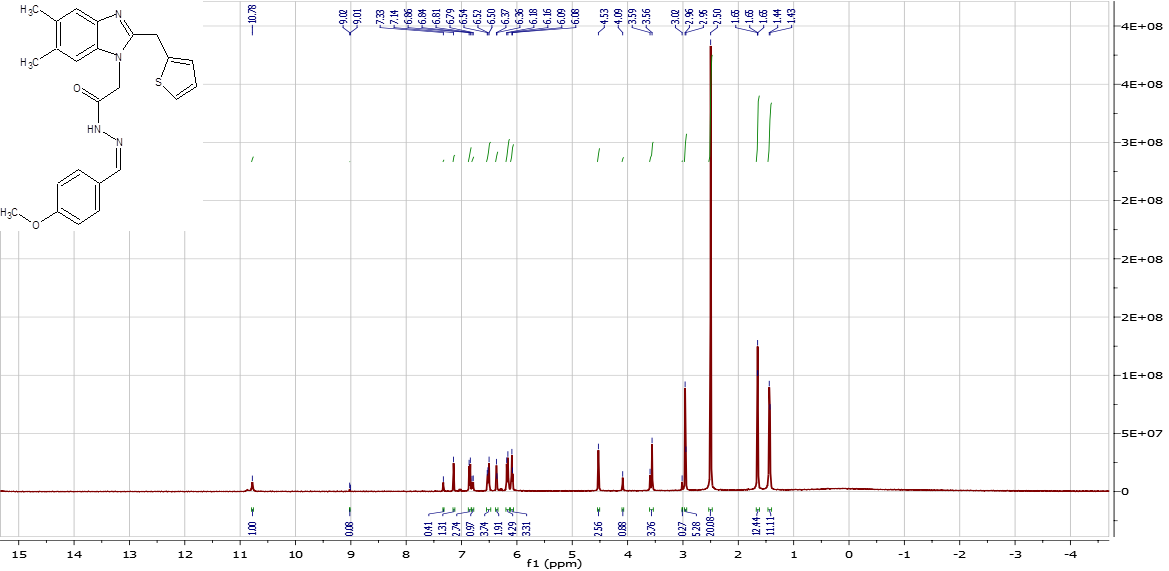


5c Compound ^1^H NMR Spectrum, 400 MHz, DMSO-d6


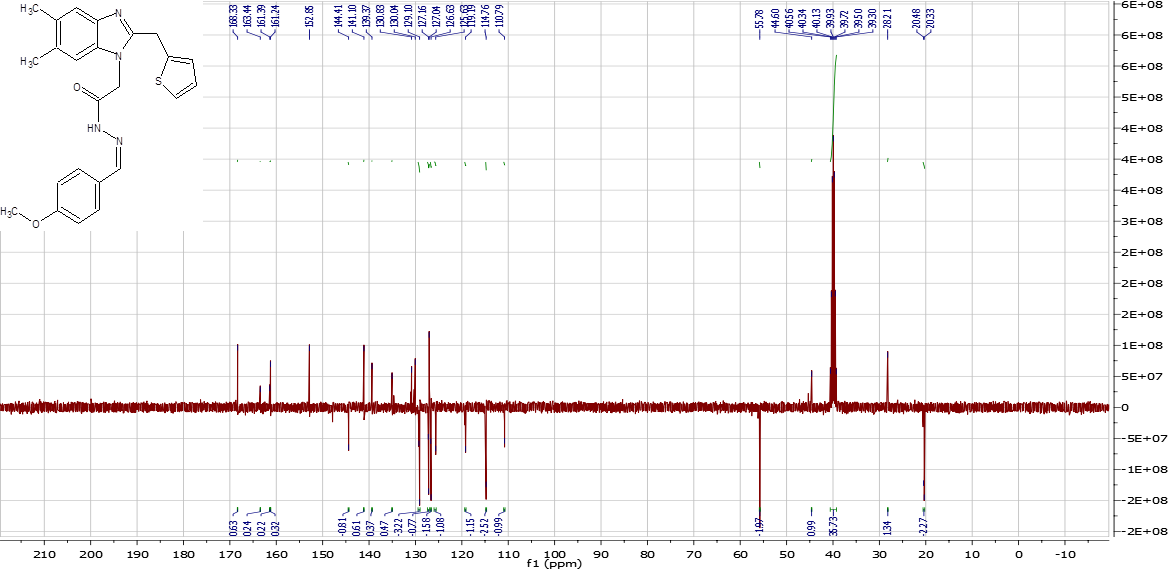


5c Compound ^13^C APT NMR Spectrum, 100 MHz, DMSO-d6

1 Compound ^1^H NMR Spectrum, 100 MHz, DMSO-d6

1 Compound ^1^H NMR Spectrum, 100 MHz, DMSO-d6

1 Compound ^1^H NMR Spectrum, 100 MHz, DMSO-d6

1 Compound ^1^H NMR Spectrum, 100 MHz, DMSO-d6

1 Compound ^1^H NMR Spectrum, 100 MHz, DMSO-d6

1 Compound ^1^H NMR Spectrum, 100 MHz, DMSO-d6

1 Compound ^1^H NMR Spectrum, 100 MHz, DMSO-d6

1 Compound ^1^H NMR Spectrum, 100 MHz, DMSO-d6

1 Compound ^1^H NMR Spectrum, 100 MHz, DMSO-d6

1 Compound ^1^H NMR Spectrum, 100 MHz, DMSO-d6

1 Compound ^1^H NMR Spectrum, 100 MHz, DMSO-d6

1 Compound ^1^H NMR Spectrum, 100 MHz, DMSO-d6

1 Compound ^1^H NMR Spectrum, 100 MHz, DMSO-d6

1 Compound ^1^H NMR Spectrum, 100 MHz, DMSO-d6

1 Compound ^1^H NMR Spectrum, 100 MHz, DMSO-d6

1 Compound ^1^H NMR Spectrum, 100 MHz, DMSO-d6

1 Compound ^1^H NMR Spectrum, 100 MHz, DMSO-d6

1 Compound ^1^H NMR Spectrum, 100 MHz, DMSO-d6

1 Compound ^1^H NMR Spectrum, 100 MHz, DMSO-d6

1 Compound ^1^H NMR Spectrum, 100 MHz, DMSO-d6

1 Compound ^1^H NMR Spectrum, 100 MHz, DMSO-d6

1 Compound ^1^H NMR Spectrum, 100 MHz, DMSO-d6

1 Compound ^1^H NMR Spectrum, 100 MHz, DMSO-d6

1 Compound ^1^H NMR Spectrum, 100 MHz, DMSO-d6

1 Compound ^1^H NMR Spectrum, 100 MHz, DMSO-d6

1 Compound ^1^H NMR Spectrum, 100 MHz, DMSO-d6

1 Compound ^1^H NMR Spectrum, 100 MHz, DMSO-d6

1 Compound ^1^H NMR Spectrum, 100 MHz, DMSO-d6

1 Compound ^1^H NMR Spectrum, 100 MHz, DMSO-d6

1 Compound ^1^H NMR Spectrum, 100 MHz, DMSO-d6

1 Compound ^1^H NMR Spectrum, 100 MHz, DMSO-d6


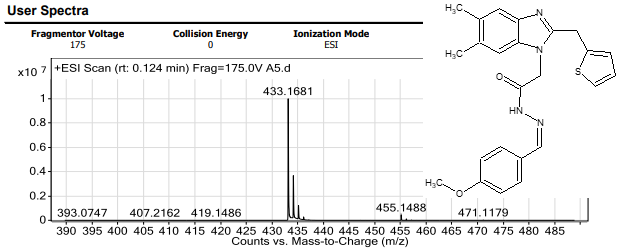


5c Compound LC/TOF-MS Spectrum

1 Compound Kütle Spectrum

1 Compound Kütle Spectrum

1 Compound Kütle Spectrum

1 Compound Kütle Spectrum

1 Compound Kütle Spectrum

1 Compound Kütle Spectrum

1 Compound Kütle Spectrum

1 Compound Kütle Spectrum

1 Compound Kütle Spectrum

1 Compound Kütle Spectrum

1 Compound Kütle Spectrum

1 Compound Kütle Spectrum

1 Compound Kütle Spectrum

1 Compound Kütle Spectrum

1 Compound Kütle Spectrum

1 Compound Kütle Spectrum

1 Compound Kütle Spectrum

1 Compound Kütle Spectrum

1 Compound Kütle Spectrum

1 Compound Kütle Spectrum

1 Compound Kütle Spectrum

1 Compound Kütle Spectrum

1 Compound Kütle Spectrum

1 Compound Kütle Spectrum

1 Compound Kütle Spectrum

1 Compound Kütle Spectrum

1 Compound Kütle Spectrum

1 Compound Kütle Spectrum

1 Compound Kütle Spectrum

1 Compound Kütle Spectrum

1 Compound Kütle Spectrum


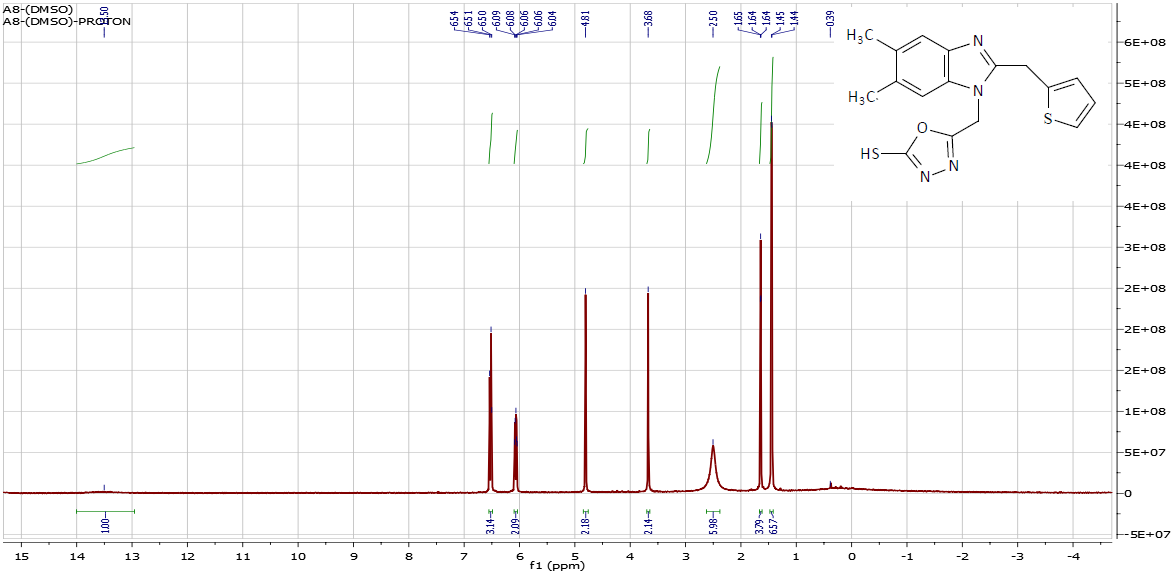


6 Compound ^1^H NMR Spectrum, 400 MHz, DMSO-d6


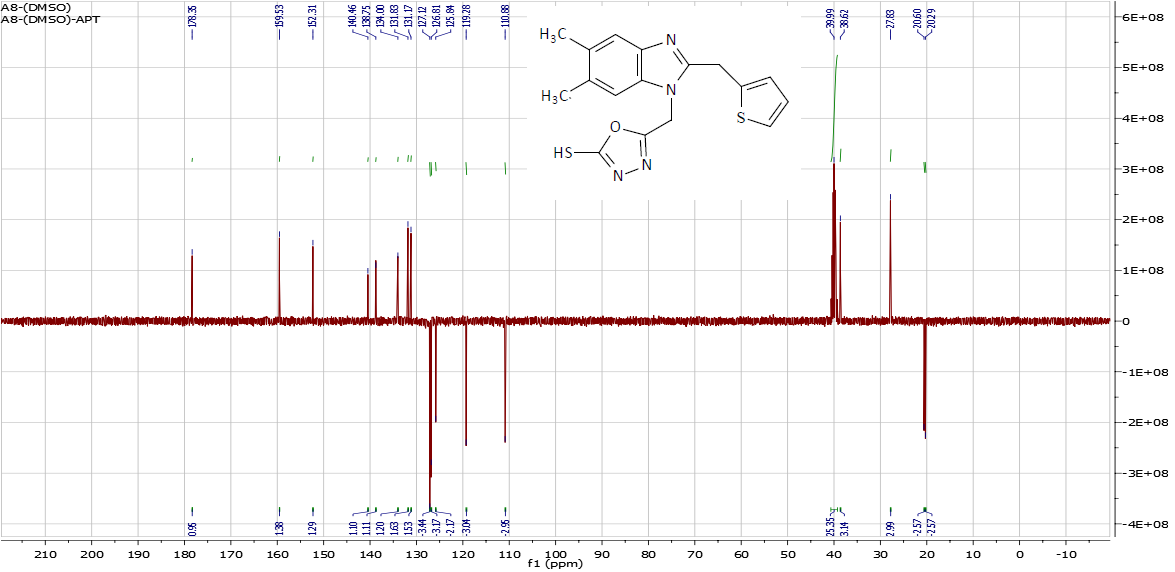


6 Compound ^13^C APT NMR Spectrum, 100 MHz, DMSO-d6

1 Compound ^1^H NMR Spectrum, 100 MHz, DMSO-d6

1 Compound ^1^H NMR Spectrum, 100 MHz, DMSO-d6

1 Compound ^1^H NMR Spectrum, 100 MHz, DMSO-d6

1 Compound ^1^H NMR Spectrum, 100 MHz, DMSO-d6

1 Compound ^1^H NMR Spectrum, 100 MHz, DMSO-d6

1 Compound ^1^H NMR Spectrum, 100 MHz, DMSO-d6

1 Compound ^1^H NMR Spectrum, 100 MHz, DMSO-d6

1 Compound ^1^H NMR Spectrum, 100 MHz, DMSO-d6

1 Compound ^1^H NMR Spectrum, 100 MHz, DMSO-d6

1 Compound ^1^H NMR Spectrum, 100 MHz, DMSO-d6

1 Compound ^1^H NMR Spectrum, 100 MHz, DMSO-d6

1 Compound ^1^H NMR Spectrum, 100 MHz, DMSO-d6

1 Compound ^1^H NMR Spectrum, 100 MHz, DMSO-d6

1 Compound ^1^H NMR Spectrum, 100 MHz, DMSO-d6

1 Compound ^1^H NMR Spectrum, 100 MHz, DMSO-d6

1 Compound ^1^H NMR Spectrum, 100 MHz, DMSO-d6

1 Compound ^1^H NMR Spectrum, 100 MHz, DMSO-d6

1 Compound ^1^H NMR Spectrum, 100 MHz, DMSO-d6

1 Compound ^1^H NMR Spectrum, 100 MHz, DMSO-d6

1 Compound ^1^H NMR Spectrum, 100 MHz, DMSO-d6

1 Compound ^1^H NMR Spectrum, 100 MHz, DMSO-d6

1 Compound ^1^H NMR Spectrum, 100 MHz, DMSO-d6

1 Compound ^1^H NMR Spectrum, 100 MHz, DMSO-d6

1 Compound ^1^H NMR Spectrum, 100 MHz, DMSO-d6

1 Compound ^1^H NMR Spectrum, 100 MHz, DMSO-d6

1 Compound ^1^H NMR Spectrum, 100 MHz, DMSO-d6

1 Compound ^1^H NMR Spectrum, 100 MHz, DMSO-d6

1 Compound ^1^H NMR Spectrum, 100 MHz, DMSO-d6

1 Compound ^1^H NMR Spectrum, 100 MHz, DMSO-d6

1 Compound ^1^H NMR Spectrum, 100 MHz, DMSO-d6

1 Compound ^1^H NMR Spectrum, 100 MHz, DMSO-d6


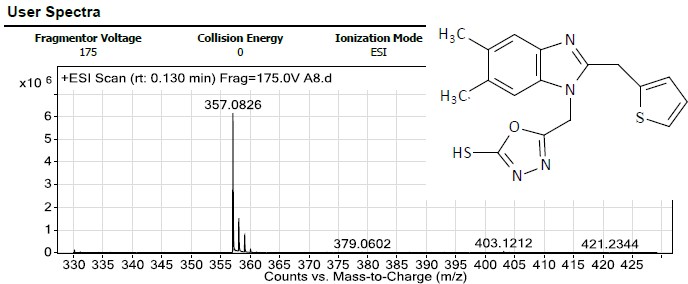


6 Compound LC/TOF-MS Spectrum

1 Compound Kütle Spectrum

1 Compound Kütle Spectrum

1 Compound Kütle Spectrum

1 Compound Kütle Spectrum

1 Compound Kütle Spectrum

1 Compound Kütle Spectrum

1 Compound Kütle Spectrum

1 Compound Kütle Spectrum

1 Compound Kütle Spectrum

1 Compound Kütle Spectrum

1 Compound Kütle Spectrum

1 Compound Kütle Spectrum

1 Compound Kütle Spectrum

1 Compound Kütle Spectrum

1 Compound Kütle Spectrum

1 Compound Kütle Spectrum

1 Compound Kütle Spectrum

1 Compound Kütle Spectrum

1 Compound Kütle Spectrum

1 Compound Kütle Spectrum

1 Compound Kütle Spectrum

1 Compound Kütle Spectrum

1 Compound Kütle Spectrum

1 Compound Kütle Spectrum

1 Compound Kütle Spectrum

1 Compound Kütle Spectrum

1 Compound Kütle Spectrum

1 Compound Kütle Spectrum

1 Compound Kütle Spectrum

1 Compound Kütle Spectrum

1 Compound Kütle Spectrum
